# The Biophysical Basis for Karyopherin-Dependent Ebola Virus VP24 Nuclear Transport

**DOI:** 10.3390/v17081051

**Published:** 2025-07-28

**Authors:** Junjie Zhao, Bojie Zhang, Olivia Vogel, Benjamin W. Walker, Leonard W. Ma, Nicole D. Wagner, Christopher F. Basler, Daisy W. Leung, Michael L. Gross, Gaya K. Amarasinghe

**Affiliations:** 1 Department of Pathology & Immunology, Washington University School of Medicine, St. Louis, MO 63110, USA; 2 Department of Medicine, Washington University School of Medicine, St. Louis, MO 63110, USAnicole.wagner@wustl.edu (N.D.W.); 3 Department of Chemistry, Washington University in St. Louis, St. Louis, MO 63130, USA; zhang.b@wustl.edu; 4 Department of Microbiology, Icahn School of Medicine at Mount Sinai, New York, NY 10029, USA; olivia.vogel@mssm.edu (O.V.); chris.basler@mssm.edu (C.F.B.)

**Keywords:** Ebola virus, nucleocytoplasmic trafficking, hydrogen-deuterium exchange mass spectrometry (HDX-MS), KPNA, VP24

## Abstract

Nucleocytoplasmic trafficking is a highly regulated process that allows the cell to control the partitioning of proteins and nucleic acids between the cytosolic and nuclear compartments. The Ebola virus minor matrix protein VP24 (eVP24) hijacks this process by binding to a region on the NPI-1 subfamily of karyopherin alpha (KPNA) nuclear importers. This region overlaps with the activated transcription factor STAT1 binding site on KPNAs, preventing STAT1 nuclear localization and activation of antiviral gene transcription. However, the molecular interactions of eVP24-KPNA5 binding that lead to the nuclear localization of eVP24 remain poorly characterized. Here, we show that trafficking of eVP24 into the nucleus by KPNA5 requires simultaneous binding of cargo. We also describe the conformational dynamics of KPNA5 and interactions with eVP24 and cargo nuclear localization sequences (NLS) using biophysical approaches. Our results reveal that eVP24 binding to KPNA5 does not impact cargo NLS binding to KPNA5, indicating that simultaneous binding of both cellular cargo and eVP24 to KPNA5 is likely required for nuclear trafficking. Together, these results provide a biophysical basis for how Ebola virus VP24 protein gains access to the nucleus during Ebola virus infection.

## 1. Introduction

Ebola virus (EBOV) is the causative agent of periodic outbreaks of Ebola virus disease (EVD) that is characterized by severe hemorrhagic fever and associated with high case fatality rates [1]. The largest Ebola outbreak on record started in Guinea in 2014 and spread to Sierra Leone and Liberia, with a total of more than 28,000 reported cases and 11,000 deaths [1]. Currently, there are limited FDA-approved vaccines and targeted therapeutics available for EVD. Thus, defining key molecular mechanisms used by the virus to circumvent host immune responses remains a critical need to better understand viral pathogenesis and to identify new targets for therapeutic development.

The cellular cytoplasm and nucleoplasm are spatially separated by the nuclear envelope. This separation is critical for compartmentalization of different cellular components and functions, including cellular transcription and replication. Key signaling macromolecules are actively transported through the nuclear pore complex (NPC), facilitated by protein transporters, including importins and exportins. The importins include karyopherin α (KPNA, also known as importin α) and karyopherin β (KPNB, also known as importin β). KPNA contains an N-terminal importin β binding domain (IBB) and a C-shaped domain containing 10 armadillo repeats (ARM), followed by an unstructured C-terminal region [2,3]. In the absence of cargos, the IBB domain of KPNA has a short classical nuclear localization sequence (cNLS)-like motif that binds to the major binding site (ARMS 2–4; residues 121–247) in an autoinhibitory manner [2,4] and a minor binding site on ARMS 7–8 (residues 331–417). Structures of yeast KPNA with SV40 T antigen NLS and human c-myc transcription factor NLS show a large binding interface at the major binding site (ARMs 2–4; residues 121–247) and a minor binding site (ARMs 7–8; residues 331–417) on KPNA [5,6]. The minor binding site likely provides a binding pocket for a bpNLS rather than being a second site for mpNLS under physiological conditions [5,6]. Proteins that enter the nucleus contain nuclear localization sequences (NLSs) that are recognized by KPNAs. Classical NLS (cNLS) contain one (monopartite, mpNLS) or two (bipartite, bpNLS) clusters of basic amino acid residues [7,8]. The best characterized mpNLS is from the SV40 large T antigen, and bpNLS is from nucleoplasmin. Cargos containing mpNLS or bpNLS bind to KPNA, forming cargo–KPNA–KPNB complexes [3,9]. The non-classical NLSs (ncNLS) lack the basic residue clusters, and their sequences are harder to define [7]. The ncNLS cargoes can be imported by KPNB directly (e.g., parathyroid hormone-related protein, PTHrP [10]) or by binding with KPNA of the KPNA–KPNB heterodimer (e.g., STAT1 [11]).

Ebola viral protein 24 (eVP24) is a minor matrix protein that has multiple functional roles in virus assembly [12,13] and interferon (IFN) antagonism. Early studies suggested that eVP24 is localized to the plasma membrane and perinuclear region of both eVP24-transfected cells and Ebola virus-infected cells [14]. eVP24 is thought to facilitate the viral nucleocapsid formation through interactions with Ebola nucleoprotein (eNP) and viral protein 35 (eVP35) along with viral protein 30 (eVP30) [15,16,17,18]. Recent studies suggest that eVP24 may be involved in genome condensation and packaging with eNP [13,18,19,20]. Furthermore, eVP24 has a critical role in modulating host immune responses like eVP35 [21,22,23,24,25,26]. Prior studies showed that cells infected with EBOV are refractory to IFN treatment [27]. Moreover, overexpression of eVP24 suppresses IFN α/β signaling and inhibits antiviral gene expression by preventing the nuclear accumulation of activated signal transducers and activators of transcription 1 (STAT1) transcription factor [25]. Further examination revealed that eVP24 interacts with the NPI-1 subfamily of nuclear transport KPNAs, including karyopherin α1 (KPNA1; importin α5), KPNA5 (importin α6), and KPNA6 (importin α7) [26,28,29,30,31,32]. Biochemical and structural analysis of the KPNA5–eVP24 interaction revealed that eVP24 binds to C-terminal ARMs of KPNA5 and prevents activated STAT1 from binding, thereby preventing STAT1 translocation into the nucleus and upregulation of IFN stimulated gene (ISG) expression [25,28,30,31,32,33].

Although our prior studies raised the possibility that eVP24 can translocate into the nucleus by binding to KPNA5, the molecular basis for this activity was unknown. In this study, we provide a molecular framework to further define interactions between eVP24 and KPNA5 and describe how this interaction is modulated by cargo-bearing cNLS through a series of biophysical studies. Specifically, we show in cell-based studies that eVP24 can colocalize into the nucleus with KPNA5 but that binding to the C-terminal ARMs of KPNA5 is not sufficient for this process and that the N-terminal ARMs of KPNA5 are required. We also used mass spectrometry (MS) methods to map the interactions and in vitro studies to characterize and validate the interactions between KPNA5–eVP24, KPNA5–cNLS, and the tri-component eVP24–KPNA–cNLS complex. To this end, we show by hydrogen–deuterium exchange (HDX) and native MS that the cNLS peptides from cargos bind to KPNA5 via either the major and/or the minor binding sites, whereas eVP24 binds to the C-terminal ARMs of KPNA5. These binding events are independent and can occur simultaneously. The autoinhibited nature of KPNA and the need for NLS engagement to release autoinhibition via disengagement of the inhibitory IBB peptide also suggest that eVP24 nuclear import is dependent on simultaneous cNLS-containing cargo binding to KPNA5. Taken together, these studies provide additional insights into eVP24 nuclear localization and reveal another regulatory layer of activity by the multifunctional eVP24 protein.

## 2. Material and Methods

### 2.1. Protein Constructs and Peptides

Human KPNA5 aa 66–508 (accession number NM_001366304) was expressed as a fusion protein with a maltose binding protein (MBP) and a His-tag in *E. coli* BL21 (DE3) cells. The fusion proteins were purified by using a series of chromatographic columns and digested with TEV protease to remove the tags prior to size-exclusion chromatography as described previously [28]. Protein molecular weights were verified by liquid chromatography–mass spectrometry (LC–MS). The purified proteins were flash frozen with liquid nitrogen, stored at −80 °C, and thawed on ice prior to use. Expression and purification of Ebola VP24 aa 11–237 (accession number AAD14588) were performed as described previously [28].

The monopartite NLS peptide (mpNLS) from the SV40 large T antigen (STPPKKKRKVE) and the bipartite NLS (bpNLS) from the nucleoplasmin (KRPAATKKAGQAKKKK) [34] were synthesized by GenScript (Piscataway, NJ, USA) with N-terminal fluorescein labels. Peptides were reconstituted with water or 1X PBS and stored at −80 °C until use.

### 2.2. Immunofluorescence Imaging

HEK-293T cells were transfected with pCAGGS-HA-eVP24 and pCAGGS-FLAG-KPNA5 (aa 1–539), pCAGGS-FLAG-KPNA5C (aa 308–509), or pCAGGS-luciferase as a control. Twenty-four hours post-transfection, cells were fixed with 4% paraformaldehyde in PBS at room temperature (RT) for 25 min, permeabilized with PBS containing 0.1% *v*/*v* Triton X-100 (PBS-T) at RT for 3 min, and blocked with 5% FBS in PBS-T at RT for 1 h. Coverslips were incubated with rabbit anti-FLAG antibody (Sigma, St. Louis, MO, USA) and mouse anti-HA antibody (Sigma, St. Louis, MO, USA) at RT for 2 h. Then, coverslips were incubated with donkey anti-rabbit antibody conjugated with AlexaFluor 647 (Invitrogen, Waltham, MA, USA), donkey anti-mouse antibody conjugated with AlexaFluor 488 (Invitrogen, Waltham, MA, USA), and DAPI (2 μg/mL) at RT for 1 h. Coverslips were mounted on superfrost slides by using ProLong Gold Antifade reagent (Thermo Fisher Scientific, Waltham, MA, USA). Images were obtained with an Olympus fluorescence microscope by using a 100× oil objective.

To calculate average nuclear fluorescence over average cytoplasmic fluorescence (fn/c), Huh7 cells (a generous gift from the Gordan laboratory at the University of California at San Francisco) were seeded at a density of 3 × 10^4^ on coverslips in a 24-well plate. The following day, cells were transfected with 200 ng pCAGGS-eVP24 and 300 ng of the indicated pCAGGS-FLAG-KPNA plasmids using lipofectamine2000 (Invitrogen, Waltham, MA, USA). Empty vector pCAGGS was included as a control. Cells were fixed in 4% paraformaldehyde in phosphate buffer saline (PBS) for 10 min at room temperature. Fixed cells were washed three times in PBS-CM (PBS, 1 mM CaCl_2_, and 1 mM MgCl_2_) and then incubated in PBG (PBS, 0.5% BSA, and 0.15% glycine) for 15 min. Cells were permeabilized in 0.1% Triton X-100 in PBG for 10 min. After washing in PBS-CM to remove permeabilization buffer, the cells were blocked with 4% goat serum in PBG for 1 h at room temperature. After blocking, cells were incubated with rabbit anti-eVP24 (SinoBiological, Paoli, PA, USA) and mouse anti-Flag (Sigma, St. Louis, MO, USA) overnight at 4 °C. Cells were then incubated with anti-rabbit Alexa Fluor 488 (Thermofisher, Waltham, MA, USA) and anti-mouse Alexa Fluor 647 (Thermofisher A-21236). Following incubation with secondary antibodies, cells were stained with Hoechst 33342 trihydrochloride (Invitrogen, Waltham, MA, USA). Following PBS washes, the coverslips were mounted with ProLong^TM^ Glass Antifade Mountant (Invitrogen, Waltham, MA, USA). Representative images were acquired using the BioTek Cytation 10 Confocal Imaging Reader using the Confocal microscope at a magnification of 60×. The BioTek Cytation 10 was also used to calculate the average fn/c of eVP24 for each condition.

Parallel transfections were performed for detection of transfected proteins by Western blot. The following antibodies were used at RT for 1 h: mouse anti-HA (Sigma, Waltham, MA, USA); mouse anti-FLAG (Sigma, Waltham, MA, USA), rabbit anti-eVP24 (developed in-house), or rabbit anti-alpha-tubulin (Sigma, St. Louis, MO, USA). Blots were incubated with anti-mouse IgG, HRP conjugated (Biorad, Hercules, CA, USA) or anti-rabbit IgG, HRP conjugated (Biorad, Hercules, CA, USA) at RT for 1 h prior to detection with chemiluminescent HRP substrate (Millipore Sigma, St. Louis, MO, USA).

### 2.3. Hydrogen–Deuterium Exchange Mass Spectrometry (HDX-MS)

The protein stock solution contained 25 µM KPNA5-ΔIBB, 25 or 75 µM eVP24, and 125 µM mpNLS or bpNLS peptide. A protein stock solution of 2 µL was diluted with 18 µL PBS buffer in D_2_O (10 mM PBS, 137 mM NaCl, 2.7 mM KCl, pH 7.4) and incubated at 25 °C for various times to allow for HDX to occur. After 10 s, 30 s, 1 min, 6 min, 15 min, 1 h, and 4 h, the reaction was quenched by adding 30 µL 3 M urea with 1% TFA (pH 2.2). The mixture was then injected into a custom-built, two-valve/two-column HDX device immersed in ice for on-line pepsin digestion and peptide separation. In brief, samples were transported in the tubing by 0.1% trifluoroacetic acid at 200 µL/min and passed through a custom-built immobilized pepsin column. Resulting peptides were captured on a 2.1 × 15 mm ZORBAX Eclipse XDB C8 column (Agilent, Santa Clara, CA, USA) and washed with solvent to remove salts. The total time for sample loading and desalting was 3 min. Peptides were then eluted with a solvent gradient delivered by a gradient pump for reversed-phase separation on a 2.1 mm × 50 mm XSelect CSH C18 column (Waters, Manchester, UK). The LC gradient started with 97.5% solvent A and 2.5% solvent B and increased to 2.5% A and 97.5% B in 6 min. Solvent A was 0.1% formic acid in water, and solvent B was 0.1% formic acid in 20% water and 80% acetonitrile. The flow rate was 100 µL/min. The LC outlet was directly connected to an electrospray ionization source of an LTQ-FT Ultra mass spectrometer (Thermo Fisher Scientific, Waltham, MA, USA) to acquire precursor-ion mass spectra (MS1) at a mass resolving power of 70,000 at *m*/*z* 400. The HDX measurement was repeated in duplicate for each time point.

Prior to HDX, peptide mapping was performed to identify the peptic-peptide sequences and their retention times to be used for HDX-MS. Non-deuterated samples were prepared by diluting 2 µL protein stock containing 25 µM KPNA5-ΔIBB or 25 µM eVP24 with 18 µL PBS buffer in H_2_O. Samples were then treated by the same procedure described above and analyzed by the mass spectrometer in the MS/MS mode with collision-induced dissociation. The peptic peptides with non-specific cleavage were identified by database searching with Byonic 2.9.59 software (Protein Metrics, Cupertino, CA, USA) against a database that contains the protein sequences. The search results were combined in the Byologic 2.9.59 software (Protein Metrics, Cupertino, CA, USA). Peptide lists that contain the sequences, charges, and retention times of peptides with MS2 scores above 200 were generated for KPNA5-ΔIBB and eVP24. The peptide lists and the LC/MS HDX data were imported to the HDExaminer software 2.5.1 (Sierra Analytics, Modesto, CA, USA) to calculate the *m*/*z* shift and percent deuterium uptake for each peptide. A total of 193 peptides from KPNA5-DIBB and 50 peptides from eVP24 were identified by using Byos 2.9.59 (Protein Metrics, Cupertino, CA, USA) and validated manually. The processed results were submitted to Kingfisher HDX-MS (an open-source web-based platform, https://kingfisher.wustl.edu/, accessed on 16 June 2025) [35] for statistical analysis and validation of HDX differences using a significance limit or *p*-value of 0.02 for KPNA5 peptides and 0.05 for eVP24 peptides.

### 2.4. Native Mass Spectrometry

KPNA5 and eVP24 proteins were buffer exchanged to 150 mM ammonium acetate (pH 6.8) with Bio-Spin P6 Columns (Bio-Rad, Hercules, CA, USA). After buffer exchange, the sample solutions contained 2 µM KPNA5-ΔIBB, with or without 2 µM eVP24, 10 µM mpNLS or bpNLS peptide. The solutions were loaded onto Nanospray ES380 platinum-coated borosilicate emitters (Thermo Fisher Scientific, Waltham, MA, USA) and sprayed into an Exactive EMR mass spectrometer (Thermo Fisher Scientific, Waltham, MA, USA). The spray voltage was 1.4 kV, in-source CID energy was 20 V, HCD collision cell energy was 45 (arbitrary units), trapping gas was 5 (arbitrary units), and data acquisition time was 1 min. Mass spectra were deconvoluted by using the Intact Mass software (Protein Metrics, Cupertino, CA, USA) for molecular weight and charge state assignment.

### 2.5. Fluorescence Polarization Assay

KPNA5 and eVP24 were dialyzed to a buffer containing 100 mM HEPES and 500 µM Mg(HCOO)_2_, pH 7.0. A total of 1 µM fluorescein-Ahx-mpNLS or 0.2 µM fluorescein-Ahx-bpNLS was mixed with 45 µL of KPNA5, eVP24, or KPNA5-eVP24 complex solutions at the concentrations indicated. Fluorescence polarization was measured using a plate reader. Each sample was measured in duplicate, and each replicate was measured twice. The polarization data were fit using the Hill equation in OriginPro (OriginLab, Northampton, MA, USA). The data points with the highest KPNA5-eVP24 complex concentrations were excluded from the fitting because the protein precipitated in those samples.

## 3. Results

### 3.1. eVP24 Binding to C-Terminal ARMs of KPNA5 Is Not Sufficient for Nuclear Transport

**Figure 1:**
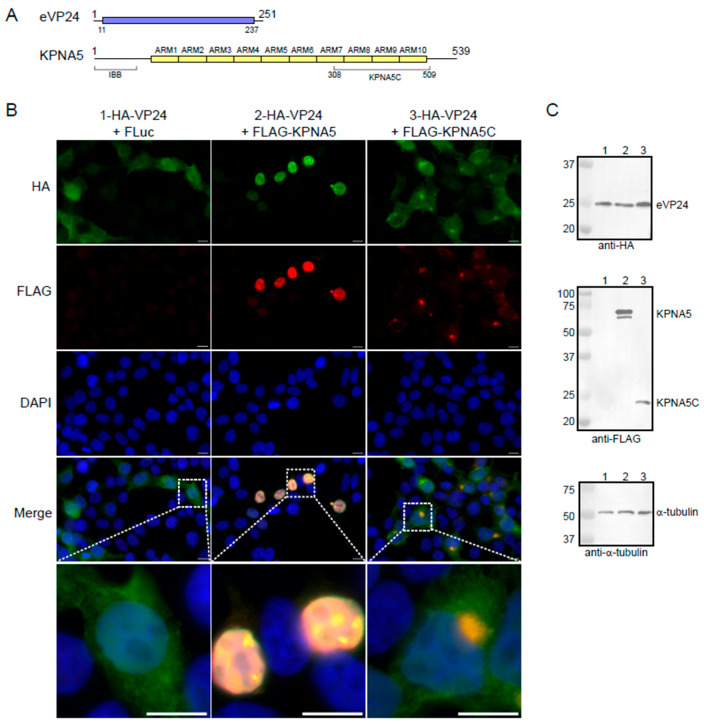
eVP24 colocalizes with KPNA5 in transfected cells. (**A**) Domain organization of eVP24 and KPNA5. (**B**) Immunofluorescence images of HEK293T cells co-transfected with HA-eVP24 (green) and FLAG-KPNA5 or FLAG-KPNA5C (red). DAPI, blue. Scale bar, 10 μm. (**C**) Western blot confirming expression of HA-eVP24 (28 kDa), FLAG-KPNA5 (62 kDa), FLAG-KPNA5C (24 kDa), and α-tubulin.

Prior studies demonstrated that eVP24 prevents activated STAT1 nuclear import by directly binding to KPNA5 [28]. We next asked if eVP24 binding to KPNA5 results in the translocation of eVP24 into the nucleus as it binds to the same C-terminal ARMs as activated STAT1. When we co-transfected 293T cells with HA-tagged eVP24 (HA-eVP24; residues 1–251) and a control plasmid, we found that eVP24 is largely distributed throughout the cytoplasm and nucleus (Figure 1A,B). Co-transfection of HA-eVP24 with FLAG-tagged KPNA5 full-length (FLAG-KPNA5; residues 1–539) resulted in the nuclear localization of both eVP24 and KPNA5. Co-transfection of HA-eVP24 with a truncated KPNA5 (FLAG-KPNA5C, residues 308–509), which lacks the N-terminal IBB domain and ARMs 1–6, showed distribution of eVP24 throughout the cytoplasm and concentration of both eVP24 and KPNA5C in perinuclear regions. Corresponding Western blots showed similar levels of expression for all proteins (Figure 1B and Figure S1). These observations indicate that eVP24 binding to the C-terminal ARMs of KPNA5 is not sufficient for nuclear translocation and that the N-terminal ARMs of KPNA5 are necessary.

**Figure 2:**
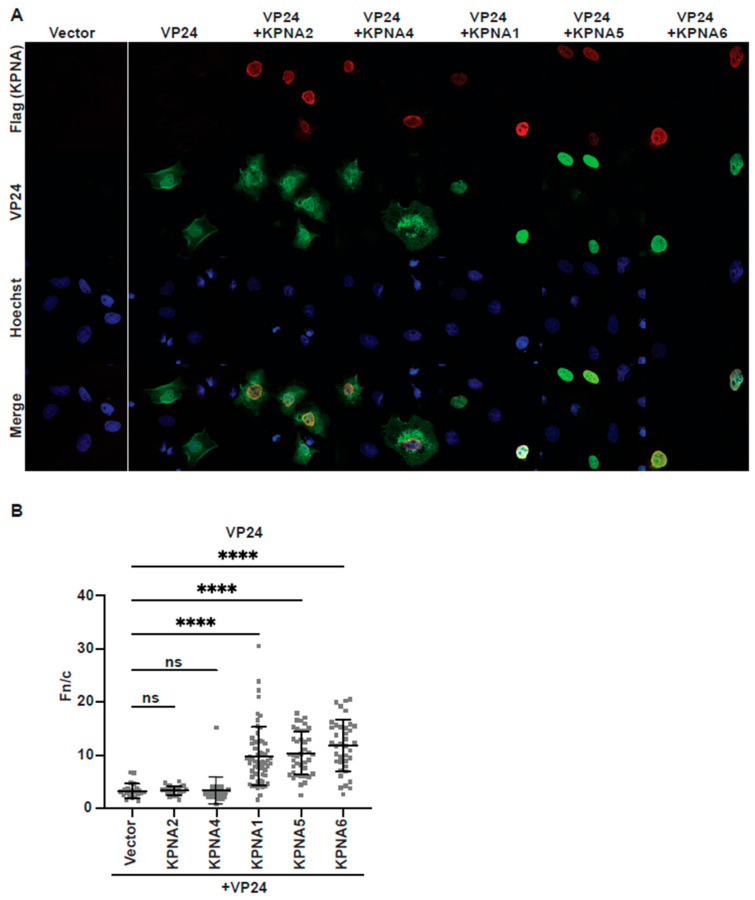
Expression of KPNA1, 5, or 6 directs eVP24 to the nucleus. (**A**) Immunofluorescence confocal images examining eVP24 (green) localization following transfection of the indicated FLAG-tagged KPNAs (red) or empty vector (Vector). (**B**) Average nuclear fluorescence over average cytoplasmic fluorescence (fn/c) of eVP24 in the presence of the indicated FLAG-tagged KPNAs or empty vector (Vector). Statistical significance was determined by one-way ANOVA, followed by Dunnett’s multiple comparison test. ns = nonsignificant, *p* ≤ 0.001 = ****.

We also tested the specificity of the eVP24–KPNA interaction for other KPNA homologs by examining eVP24 localization following co-transfection of KPNA2, KPNA4, KPNA1, KPNA5, and KPNA6 (Figure 2A,B). We calculated the average nuclear fluorescence over the average cytoplasmic fluorescence (fn/c) to quantify changes in eVP24 localization following co-transfection of different KPNA homologs (Figure 2B). Compared to expression of eVP24 alone, co-expression with KPNA1, KPNA5, and KPNA6 led to increased nuclear accumulation and decreased cytoplasmic localization of eVP24 (Figure 2A,B). In contrast, co-expression of the other KPNA family members KPNA2 and KPNA4 had little to no impact on eVP24 localization (Figure 2A,B). This is consistent with a previous study showing that eVP24 binds to KPNA1, KPNA5, and KPNA6, while KPNA2 and KPNA4 do not bind eVP24 [26].

### 3.2. mpNLS and bpNLS Peptides Bind the N-Terminal ARMs of KPNA5

To determine if cargo binding is required for the nuclear trafficking of eVP24, we evaluated binding of an mpNLS (SV40 T antigen) or bpNLS peptide (nucleoplasmin) (to represent cargo) and eVP24 to KPNA5. We conducted HDX-MS studies to define the binding interfaces of KPNA5 in solution and to characterize changes in the solvent accessibility of the KPNA5 protein backbone upon addition of NLS peptides and eVP24. The results of HDX-MS analyses on KPNA5 in complex with mpNLS and bpNLS peptides have been statistically validated and compiled into volcano plots and Woods’ plots showing several peptide regions of KPNA5 affected by the addition of mpNLS peptide, bpNLS peptide, and eVP24 (Figures S1 and S3). Each of these regions show a significant decrease in deuterium uptake consistent with protection in the bound state.

**Figure 3:**
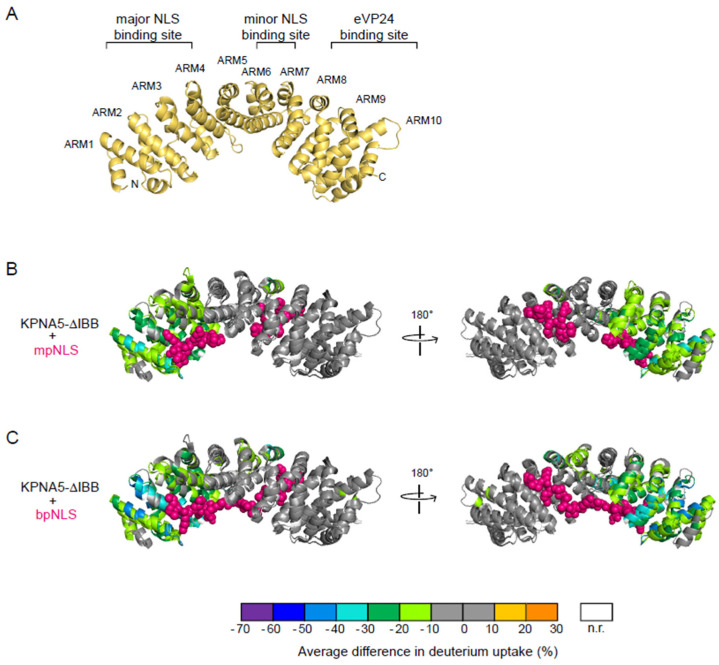
HDX-MS analyses define cNLS binding sites on KPNA5. (**A**) Structure of *apo*-KPNA (PDB 1BK5) showing the arrangement of armadillo repeats (ARM) 1–10. HDX protection on KPNA5-ΔIBB upon binding to (**B**) the SV40 large T antigen mpNLS peptide or (**C**) the nucleoplasmin bpNLS. The differences in deuterium uptake are mapped onto the structure of yeast KPNA with SV40 mpNLS (PDB 1BK6) and/or nucleoplasmin bpNLS (PDB 1EE5). NLS peptide residues are represented as pink spheres.

A crystal structure of *apo*-KPNA is presented to show the arrangement of the ARMs (Figure 3A; PDB 1BK5) [5]. Results were mapped onto the X-ray crystal structures of KPNA in complex with mpNLS (PDB 1BK6) or bpNLS (PDB 1EE5) peptides. We find that the binding of an mpNLS peptide induces large changes in solvent accessibility on KPNA5 ARMs 1–4 (Figure 3A,B, Figures S1B and S3A). This protection extends beyond the immediate interface at the major NLS binding site to the entire ARMs 1–4, except for a few residues at the N-terminus of ARM 1. These results suggest that mpNLS peptide binding reduces the structural flexibility of the helices in ARMs 1–4 and that the binding may increase the stability of KPNA5 in the KPNA5–cargo complex. In contrast, the remaining ARMs 5–10 do not show any significant differences in solvent accessibility, except in ARM7. At the minor NLS binding site, residues 330–335 on ARM7 show a more than 10% deuterium uptake decrease upon mpNLS peptide binding (Figure 3B). Residues 280, 293–298 on ARM6, and 396–402 on ARM 9 show a 9–10% decrease in HDX when the nearby non-interacting residues show less than a 6% decrease (Figure S3). These regions contain the interacting residues as identified by Conti et al. and Fontes et al. [5,6,34]. The results suggest that the mpNLS peptide binding affinity is much lower at the minor NLS binding site than at the major NLS binding site.

Next, we compared the binding of a bpNLS peptide to KPNA5 (Figure 3C, Figures S1C and S2B). Similar to HDX of the mpNLS peptide, we observe significant changes in HDX-MS results in the sequence for ARMS 1–4. This site corresponds to the major NLS binding site (Figure 3C). In contrast to the data for mpNLS, we find that the bpNLS causes more residues to show decreased HDX perturbations. These include residues near the minor NLS binding site, including residues 284–299 on ARM6; 330–335, 339–340, and 342–344 on ARM 7; 364–375 on ARM8; and 396–402 on ARM9. Overall, the decrease in HDX rates on ARMs 6–9 is also lower in magnitude than compared to ARMs 1–4, suggesting that the bpNLS peptide has a lower affinity (undergoes more rapid off rates) at the minor binding site.

### 3.3. Binding of eVP24 Is Localized to the C-Terminal ARMs of KPNA5

**Figure 4:**
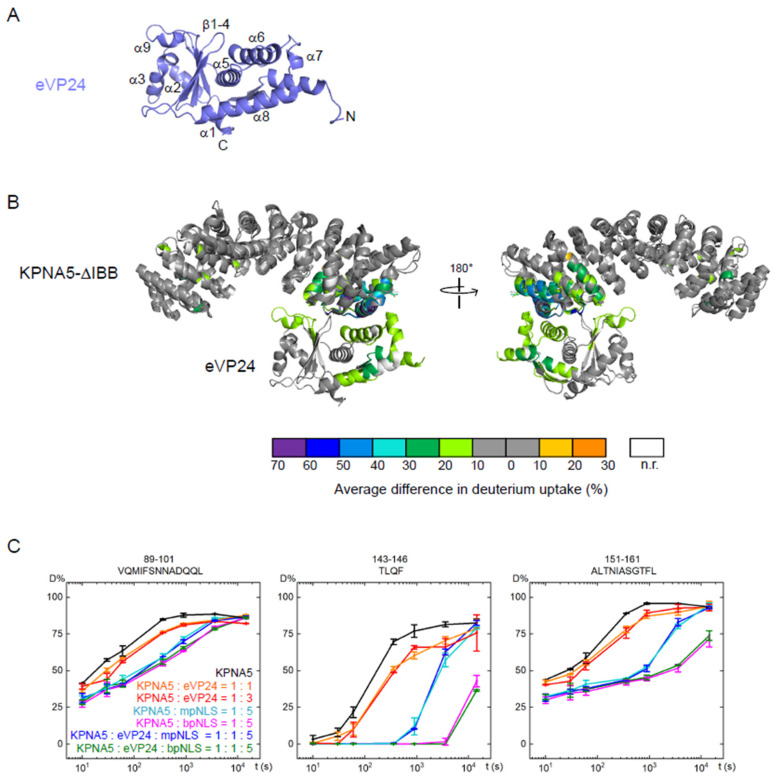
HDX-MS analyses of the eVP24–KPNA5 complex reveal protection in ARMs 8–10. (**A**) Structure of eVP24 (PDB 4M0Q) showing the arrangement of secondary structures. (**B**) HDX protection on KPNA5-ΔIBB and eVP24 upon binding. Results are mapped onto the structural alignment of the eVP24–KPNA5C (aa 308–509) complex (PDB 4U2X) with yeast KPNA (PDB 1BK6). (**C**) HDX plots of representative KPNA5 peptides showing a remote conformational change caused by eVP24 binding.

We previously described the X-ray crystal structure of eVP24 (Figure 4A) and eVP24 bound to KPNA5 ARMs 7–10 (308–509) [28,36]. Although this structure reveals the structural basis for eVP24–KPNA interaction, we did not explore the role of cargos in the context of eVP24 binding. To evaluate the impact of eVP24 binding to KPNA5-ΔIBB and to define the relationship between cNLS binding and eVP24 binding, we used HDX-MS on a variety of two- and three-component complexes. The results of HDX-MS analyses on eVP24 in complex with KPNA5 and cNLS peptides were statistically validated and compiled into volcano plots and Woods’ plots showing several peptide regions of KPNA5 affected by the addition of cNLS peptides and eVP24 (Figures S1D–F and S2C–F). Each of these regions show a significant decrease in deuterium uptake consistent with protection in the bound state.

The corresponding HDX rates reveal a large interface on KPNA5 ARMs 8–10 that is protected from deuterium uptake upon eVP24 binding to KPNA5 (Figure 4B). This observation is consistent with the X-ray crystal structure of the eVP24–KPNA5C complex [28]. The binding interface includes residues 387–402 on ARM 8, residues 432–442 on ARM 9, and residues 472–508 on ARM 10. Thus, unlike the cNLS peptides, eVP24 binding exclusively impacts the solvent exposure of residues that are directly involved in the binding interface on ARMs 8–10. For most of the peptides in these regions, the HDX rate plots of the bound state show very low deuterium uptake levels that do not converge with the plots for the unbound state after 4 h (Figure S3). These results suggest low off rates and high affinity binding between KPNA5 and eVP24, which is consistent with findings from previous studies [28,29].

In addition to the changes described above at the KPNA5 C-terminus, we also observed that there were marginal decreases in the HDX rates for peptides in ARMs 2 and 3 of KPNA5-ΔIBB upon eVP24 binding (Figure 4B,C, Figures S2C and S3). Increasing the eVP24:KPNA5-ΔIBB ratio did not cause further decreases in HDX (Figure 4C), indicating that this region is not a secondary binding site for eVP24, but rather, this perturbation represents conformational changes due to eVP24 binding at the distant ARMs 8–10. The HDX rate protection observed on KPNA5 residues 89–101, 143–146, and 151–161 upon eVP24 binding suggests that there are allosteric conformational changes occurring at the major cNLS binding site (Figure 4C). Although these differences do not meet the stringent statistical limits set for this dataset (Figure S2C), these regions are of potential interest for future investigations.

We also examined the binding interface on eVP24 (Figure 4B, Figures S4 and S5). The structure of eVP24 bound to KPNA5 ARMs 7–10 shows extensive contacts on eVP24, including helices α6, α7, and α9 and surrounding loops [28]. We identified by HDX-MS that backbone protection occurs for residues on helices α6, α7, and α9 that directly interact with KPNA5, consistent with the structure of the complex (Figure 4A,B). We also found protection on helices α1 and α8 that do not apparently participate in the direct interaction with KPNA5. These observations suggest that the overall structure of eVP24 displays less flexibility upon binding to KPNA5.

### 3.4. Simultaneous Binding of eVP24 and cNLS Peptides on KPNA5

**Figure 5:**
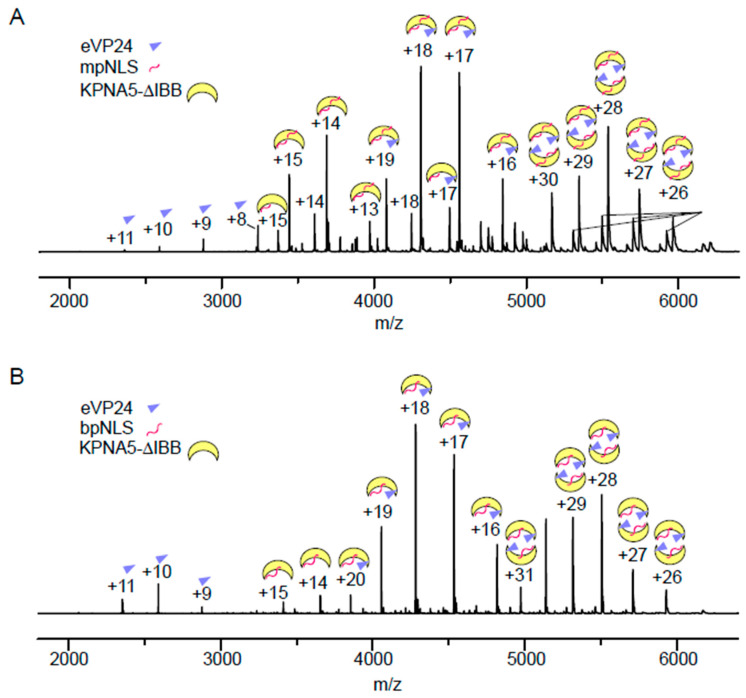
Native mass spectrometry analyses reveal the presence of cNLS–KPNA5–eVP24 complexes. Native mass spectra confirm formation of different complexes containing KPNA5-ΔIBB, eVP24, and (**A**) mpNLS or (**B**) bpNLS peptides. Spectral peaks are assigned based on their deconvoluted masses.

Given that the cNLS peptides and eVP24 occupy distinct sites on KPNA5, we next asked whether cNLS peptides and eVP24 can bind KPNA5 simultaneously or competitively. We generated samples containing eVP24 and KPNA5-ΔIBB and added either mpNLS or bpNLS peptides prior to performing high-resolution mass spectrometry under native spray conditions. Analysis of the mass spectrum revealed the formation of several complexes. For samples containing the mpNLS, the stoichiometry of eVP24:KPNA5-ΔIBB:mpNLS is 1:1:1 or 1:1:2 (Figure 5A). This indicates that both eVP24 and mpNLS bind KPNA5-ΔIBB. Furthermore, the data show that the mpNLS binds at the major and the minor NLS binding sites. We also observed dimers of eVP24:KPNA5-ΔIBB:mpNLS complexes at high *m*/*z*, which is not unexpected given prior data showing that eVP24 has the potential to dimerize [18,36,37]. Furthermore, KPNA and KPNA–NLS dimers were observed in previous studies, where the dimer interfaces are on the concave side of the C-shaped ARM domains [5,6,34]. When we examined samples containing the bpNLS peptide, we found the stoichiometry of eVP24:KPNA5-ΔIBB:bpNLS is 1:1:1 (Figure 5B), suggesting that the bpNLS peptides bind in a groove extending from the major NLS binding site to the minor NLS binding site on KPNA5. Dimers of eVP24–KPNA5–bpNLS are also observed. Altogether, our native MS results show that eVP24 binds to KPNA simultaneously as the mpNLS or bpNLS peptides that do not impact dimerization of eVP24 or KPNA5.

**Figure 6:**
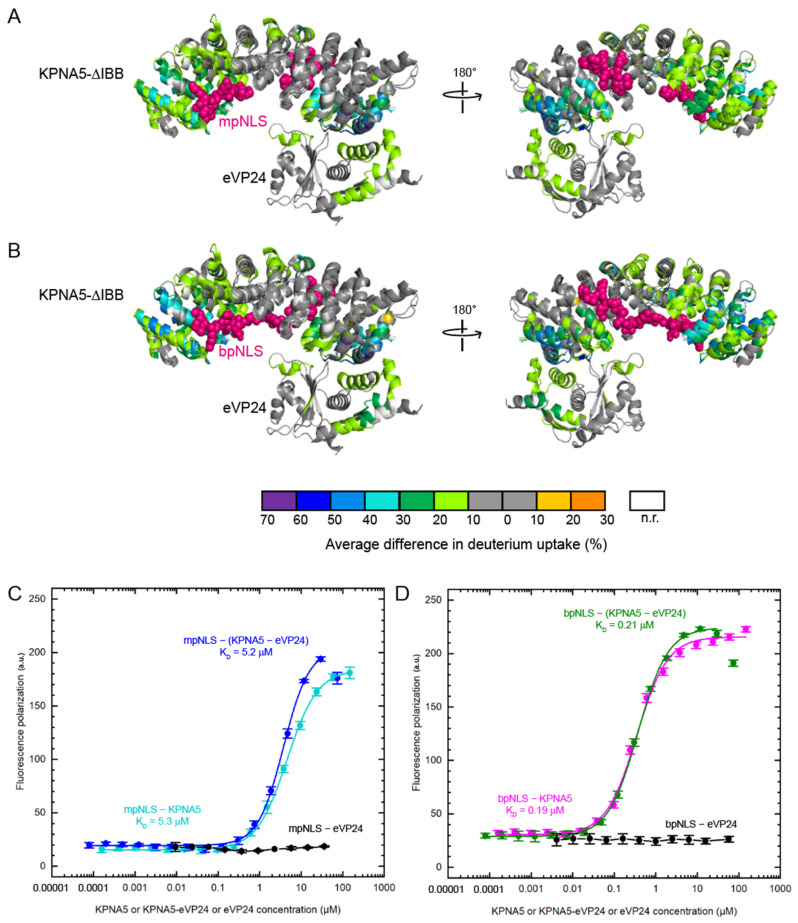
HDX-MS analyses and fluorescence polarization of cNLS–eVP24–KPNA5 complexes confirm simultaneous binding of eVP24 and cNLS peptide on KPNA5. HDX protection mapped onto the KPNA5-ΔIBB/eVP24 complex structure upon addition of eVP24 and (**A**) mpNLS or (**B**) bpNLS peptide. Results are mapped onto the structural alignment of the eVP24–KPNA5C (308–509) complex (PDB 4U2X) with yeast KPNA with SV40 mpNLS (PDB 1BK6) or yeast KPNA with nucleoplasmin bpNLS (PDB 1EE5). NLS peptides are represented as pink spheres. Fluorescence polarization assay results using fluorescein labeled (**C**) mpNLS peptide or (**D**) bpNLS peptide. The binding affinities (K_D_) are 5.3 µM for mpNLS with KPNA5 (cyan), 5.2 µM for mpNLS with KPNA5–eVP24 (blue), 0.19 µM for bpNLS with KPNA5 (pink), 0.21 µM for bpNLS with KPNA5–eVP24 (green), and not determined for mpNLS or bpNLS with eVP24 (black).

To further support the finding that eVP24 and NLS peptides can both bind KPNA5, we performed additional HDX-MS experiments of the eVP24–KPNA5–ΔIBB–NLS peptide complexes (Figure 6A,B). We find that the regions on KPNA5 that are protected from deuterium exchange are similar to those of KPNA5–mpNLS peptide (compare Figure 6A to Figure 3B), KPNA5–bpNLS peptide (compare Figure 6B to Figure 3C), and KPNA5–eVP24 individually. These comparisons confirm that both eVP24 and NLS peptides bind to KPNA5 and that binding of eVP24 and NLS peptides does not induce additional significant changes in KPNA5.

### 3.5. eVP24 Does Not Impact NLS Peptide Binding on KPNA5

Because we observed that eVP24 binding to KPNA5-ΔIBB caused some decrease in HDX on the ARMs involving the major NLS binding site, we next investigated how these conformational changes impact the NLS peptide binding. We carried out fluorescence polarization assays to measure the binding affinity between KPNA5-ΔIBB and N-terminal fluorescein labeled mpNLS and bpNLS peptides. The results show that neither the mpNLS or bpNLS peptide binds eVP24, as expected (Figure 6C,D). The mpNLS binds KPNA5-ΔIBB with higher apparent affinity (Figure 6C; *K_D_* = 5.3 µM) compared to the bpNLS peptide (Figure 6D; *K_D_* = 0.19 µM). Addition of the eVP24–KPNA5-ΔIBB complex does not change the apparent binding affinities of KPNA5 for either mpNLS (*K_D_* = 5.2 µM) or bpNLS (*K_D_* = 0.21 µM) peptides, indicating that eVP24 binding does not impact classical cargo NLS binding to KPNA5, as we showed previously [28].

## 4. Discussion

During Ebola virus infection, eVP24 blocks the STAT1 signaling pathway, preventing phosphorylated STAT1 from binding to KPNA5 and being transported into the nucleus and activating antiviral gene transcription. This blocking has clinical implications and prevents broad spectrum first-line drugs such as IFNβ from being used to limit inflammation. Thus, eVP24 targeting of STAT1 signaling provides an effective means to shut down host antiviral responses. Interestingly, eVP24 binding to KPNA5 does not prevent cellular cargoes containing a classical NLS from binding to KPNA5. In the studies described here, we find that KPNA5 alone and the KPNA5–eVP24 complex both bind to a mpNLS peptide derived from SV40 large T antigen or a bpNLS peptide derived from nucleoplasmin and with similar binding affinities. These binding results suggest that, at least for some classical NLS-containing cargo proteins, eVP24 binding does not impact cellular cargo–KPNA5 interactions, validating the potential for concurrent nucleocytoplasmic transport of both eVP24 and cargos by KPNAs.

Although the binding affinities between SV40 T antigen or nucleoplasmin NLS peptides and KPNA5 are not affected by eVP24 binding, it is possible that other cargo binding can be affected. Our HDX-MS data reveal that eVP24 binding to KPNA5 induces modest long-range conformational changes in the major NLS binding region of KPNA5, and these changes may influence cargo selectivity of the NPI-1 subfamily of importers. Possible functions include regulation of gene expression by altering the nucleocytoplasmic distribution of transcription factors or of innate immune effectors by sequestering them in cellular compartments or facilitating their transport. For example, eVP24–KPNA binding partially relocates the heterogenous nuclear ribonuclear protein complex C1/C2 (hnRNP C1/C2) from the nucleus to the cytoplasm [38], although the function and biological significance have yet to be established. In addition, eVP24 binding may recruit other cytoplasmic cargos to KPNAs. Additional studies are needed to explore these possibilities.

Our current study also confirms that cargo containing classical NLS binding is necessary for the transport of eVP24 into the nucleus. Expression of a KPNA5 construct lacking the N-terminal ARMs containing the major NLS binding site colocalizes with eVP24 but is retained in the cytoplasm. Observation of nuclear translocation of eVP24 suggests that eVP24 may have additional functions within the nucleus. This hypothesis is supported by proteomics studies that identified potential eVP24 interactions with nuclear-resident proteins [39,40]. It will be interesting to determine if eVP24 also regulates IFN responses at the level of gene expression observed in other negative strand RNA viruses [41,42,43] and contributes to the long-term impact of Ebola virus infection even after the virus is cleared in survivors of EVD. Finally, the nucleocytoplasmic trafficking of eVP24 itself may influence multiple functions of eVP24. The import of eVP24 into the nucleus can provide a method of sequestration to prevent eVP24 from prematurely facilitating nucleocapsid condensation and assembly, which has been observed in other viral systems [43]. Further studies are warranted to dissect these possibilities.

Our current study also confirms that binding of classical NLS-containing cargo is necessary for the transport of eVP24 into the nucleus. Expression of a KPNA5 construct lacking the N-terminal ARMs containing the major NLS binding site colocalizes with eVP24 but is retained in the cytoplasm. The observation of nuclear translocation of eVP24 suggests that eVP24 may have additional functions within the nucleus. This hypothesis is supported by proteomics studies that identified potential eVP24 interactions with nuclear-resident proteins [39,44]. It will be interesting to determine if eVP24 also regulates IFN responses at the level of gene expression observed in other negative strand RNA viruses [40,41,42] and contributes to the long-term impact of Ebola virus infection even after the virus is cleared in survivors of EVD. Finally, the nucleocytoplasmic trafficking of eVP24 itself may influence multiple functions of eVP24, including in nucleocapsid assembly. Localization of eVP24 into the nucleus can provide a method of sequestration to prevent eVP24 from prematurely facilitating nucleocapsid condensation and assembly, which has been observed in other viral systems [43]. Additional studies are warranted to dissect these possibilities.

## Data Availability

Available upon request.

## Supplementary Materials

The following supporting information can be downloaded at: https://www.mdpi.com/article/10.3390/v17081051/s1, Figure S1: Anti-VP24 western blot and HDX-MS showing large numbers of protected peptides for KPNA5 in different mixtures; Figure S2: A sequence aligned view of statistically validated HDX differences reveals regions of KPNA5 affected by binding; Figure S3: HDX kinetic plots for eVP24 and KPNA5; Figure S4: HDX-MS reveals large numbers of protected peptides for eVP24 in different mixtures; Figure S5: A sequence aligned view of statistically validated HDX differences reveals regions of eVP24 affected by binding.
