# Supplementary material for: The Biophysical Basis for Karyopherin-Dependent Ebola Virus VP24 Nuclear Transport
Source: Viruses. 2025 Jul 28;17(8):1051. doi: 10.3390/v17081051 (PMC12390655; doi:10.3390/v17081051)
Supplement: Supplementary file 1 [file viruses-17-01051-s001.zip › viruses-3654727-supplementary.pdf]

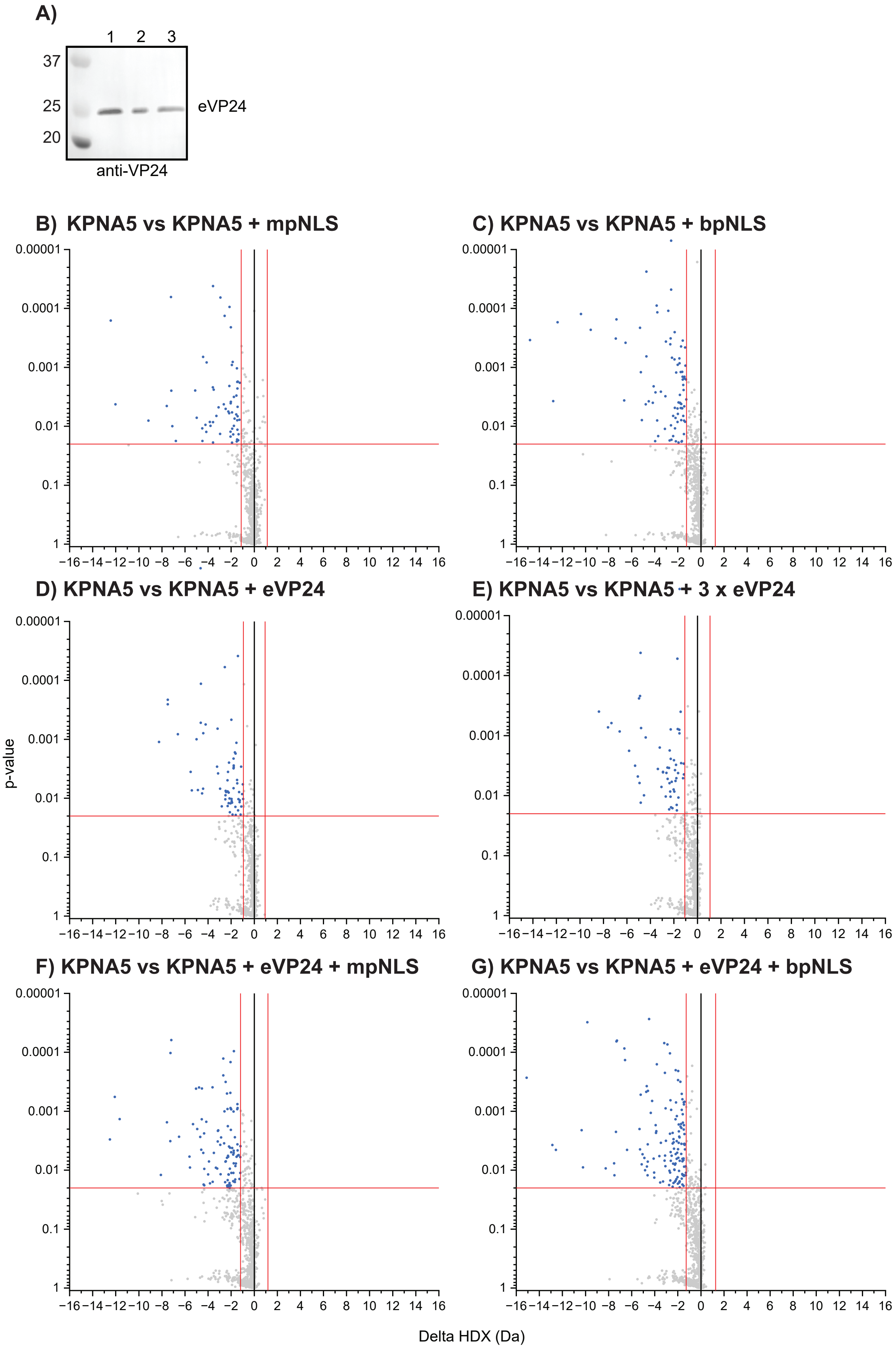

Supplementary Figure S1

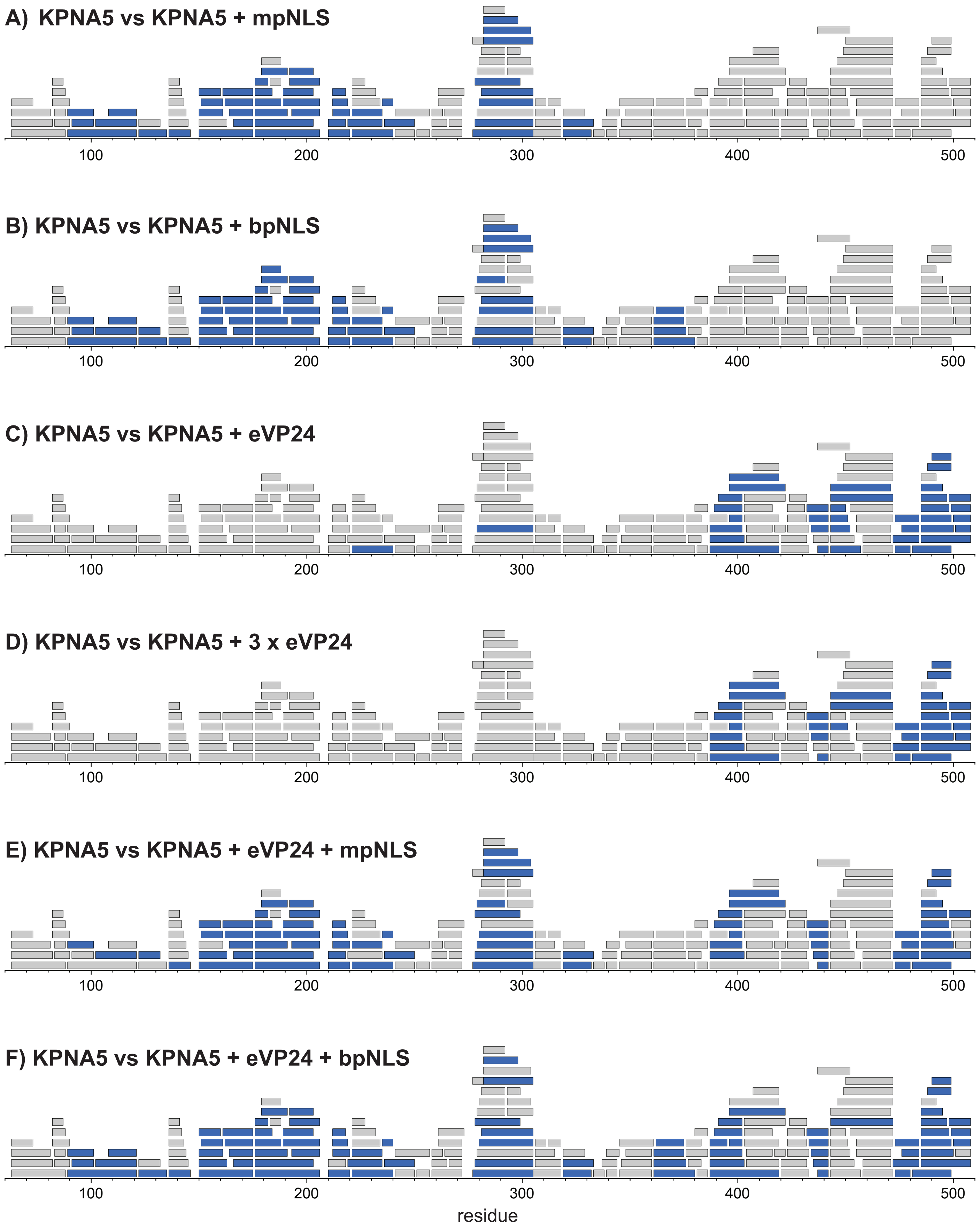

Supplementary Figure S2

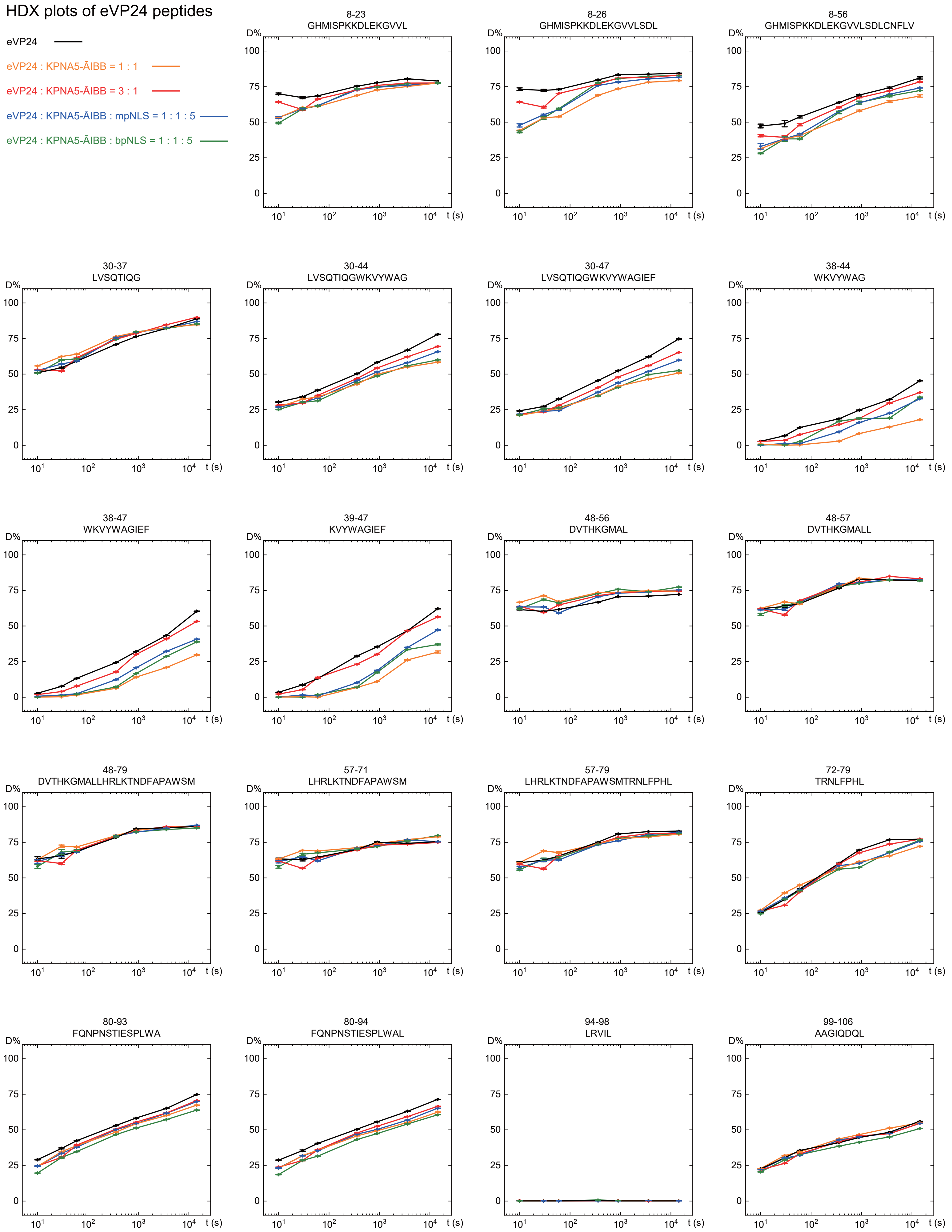

Supplementary Figure S3

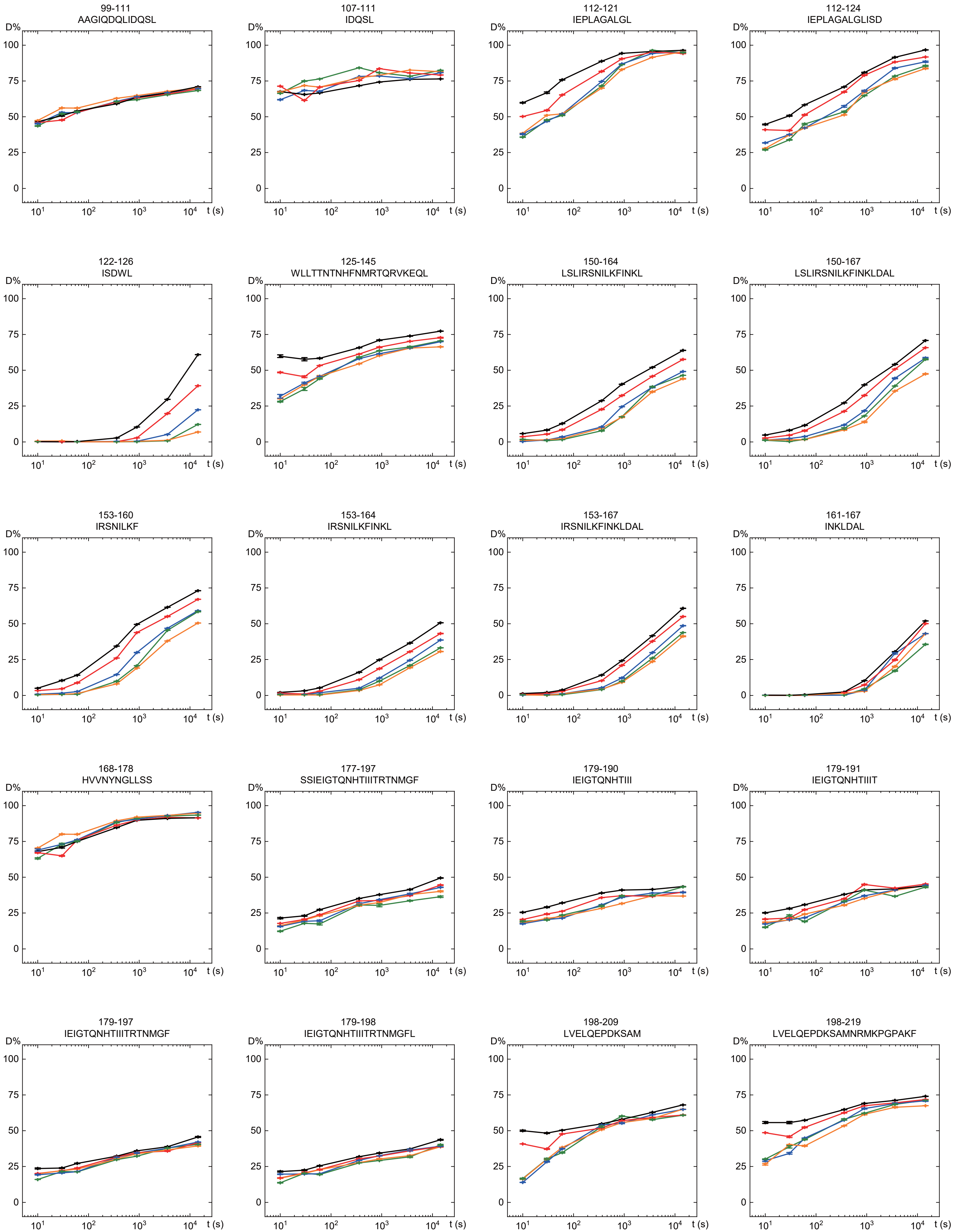

Supplementary Figure S3

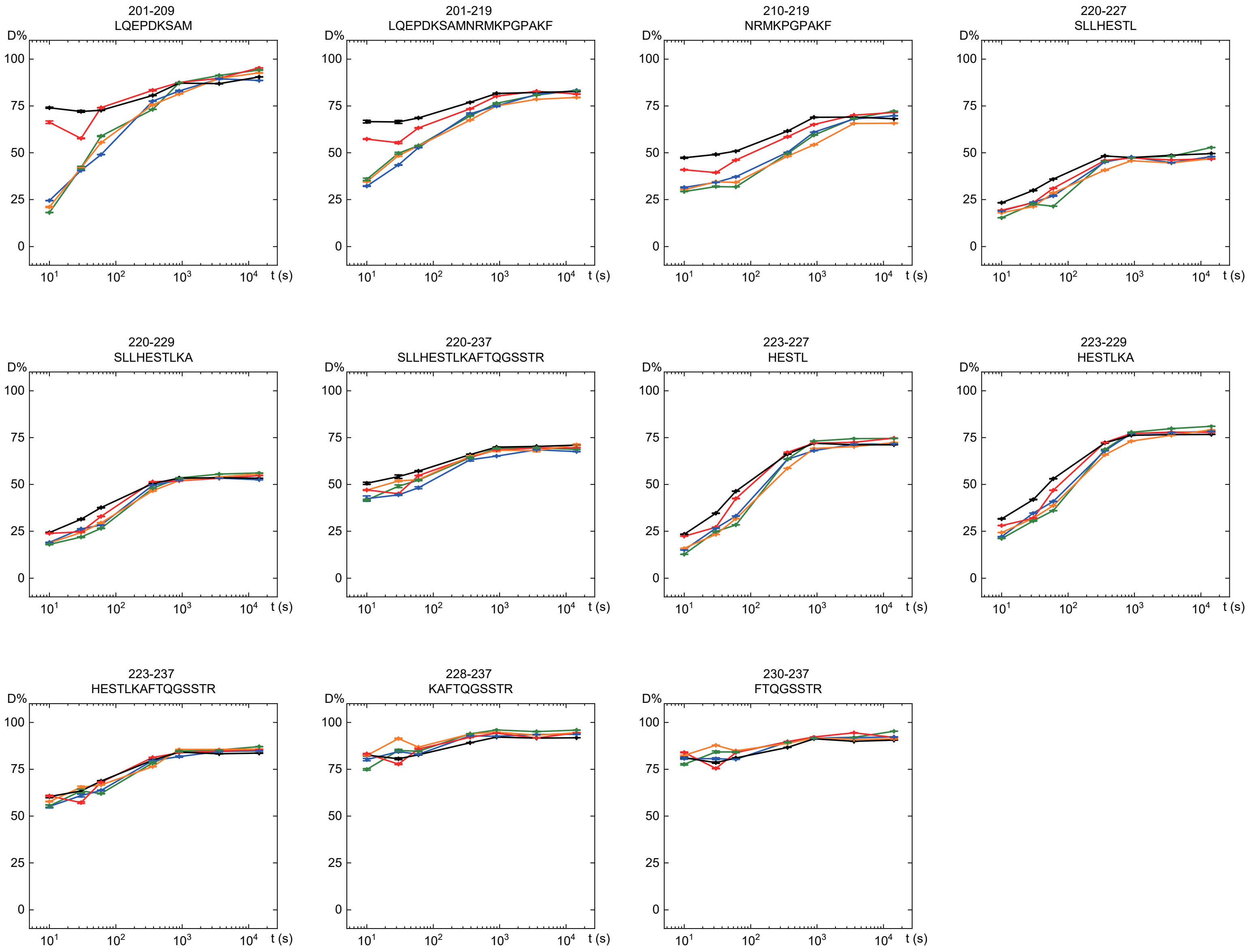

Supplementary Figure S3

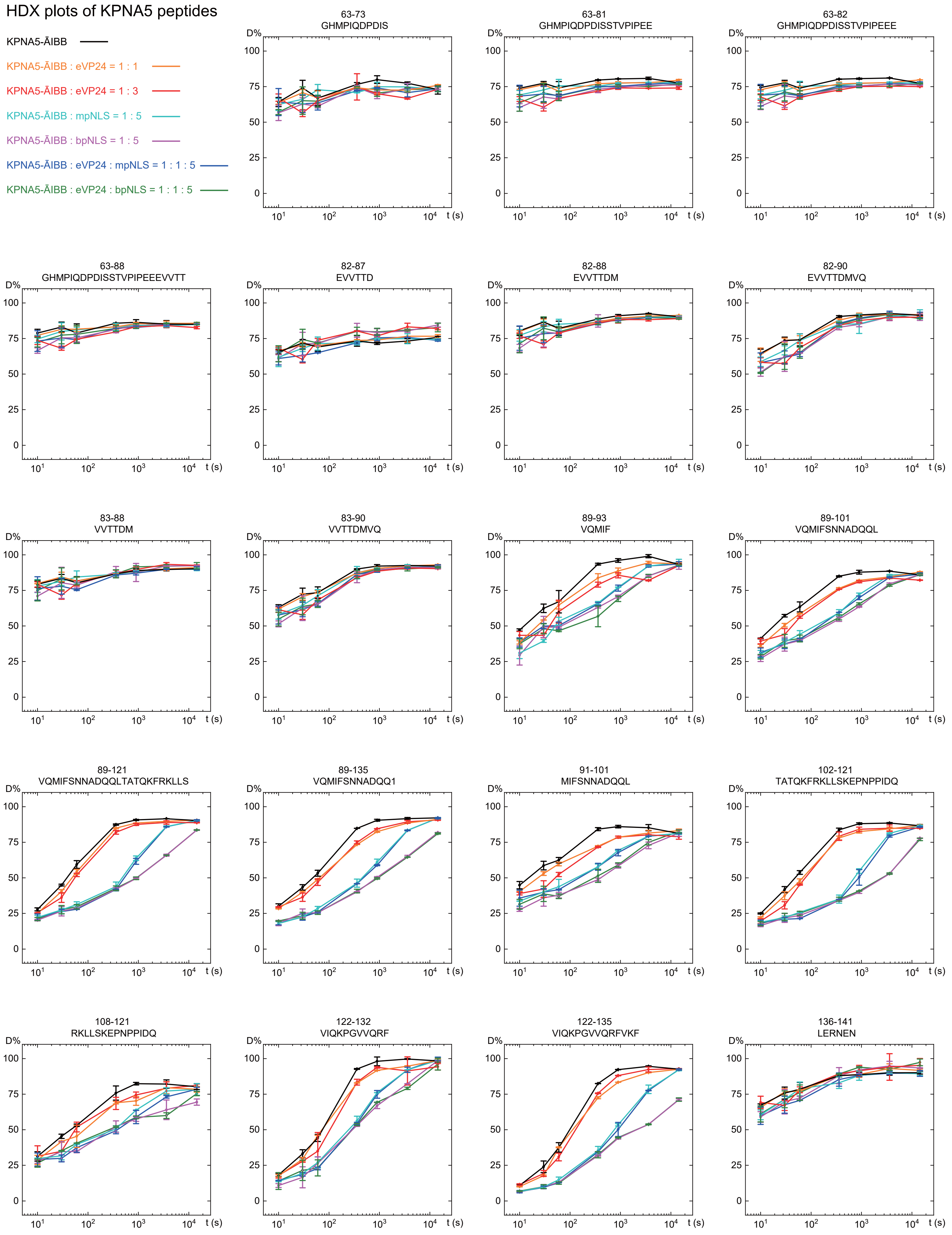

Supplementary Figure S3

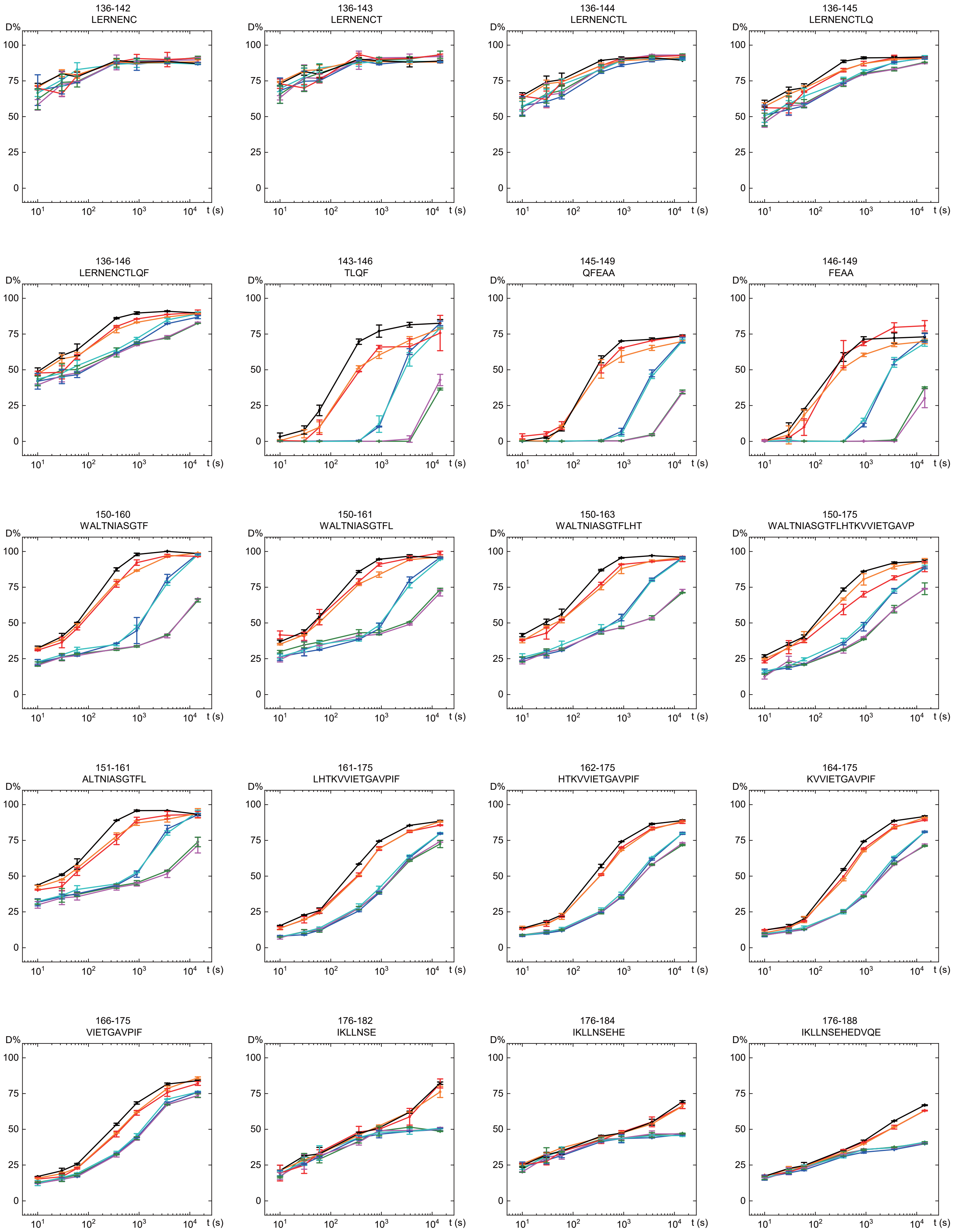

Supplementary Figure S3

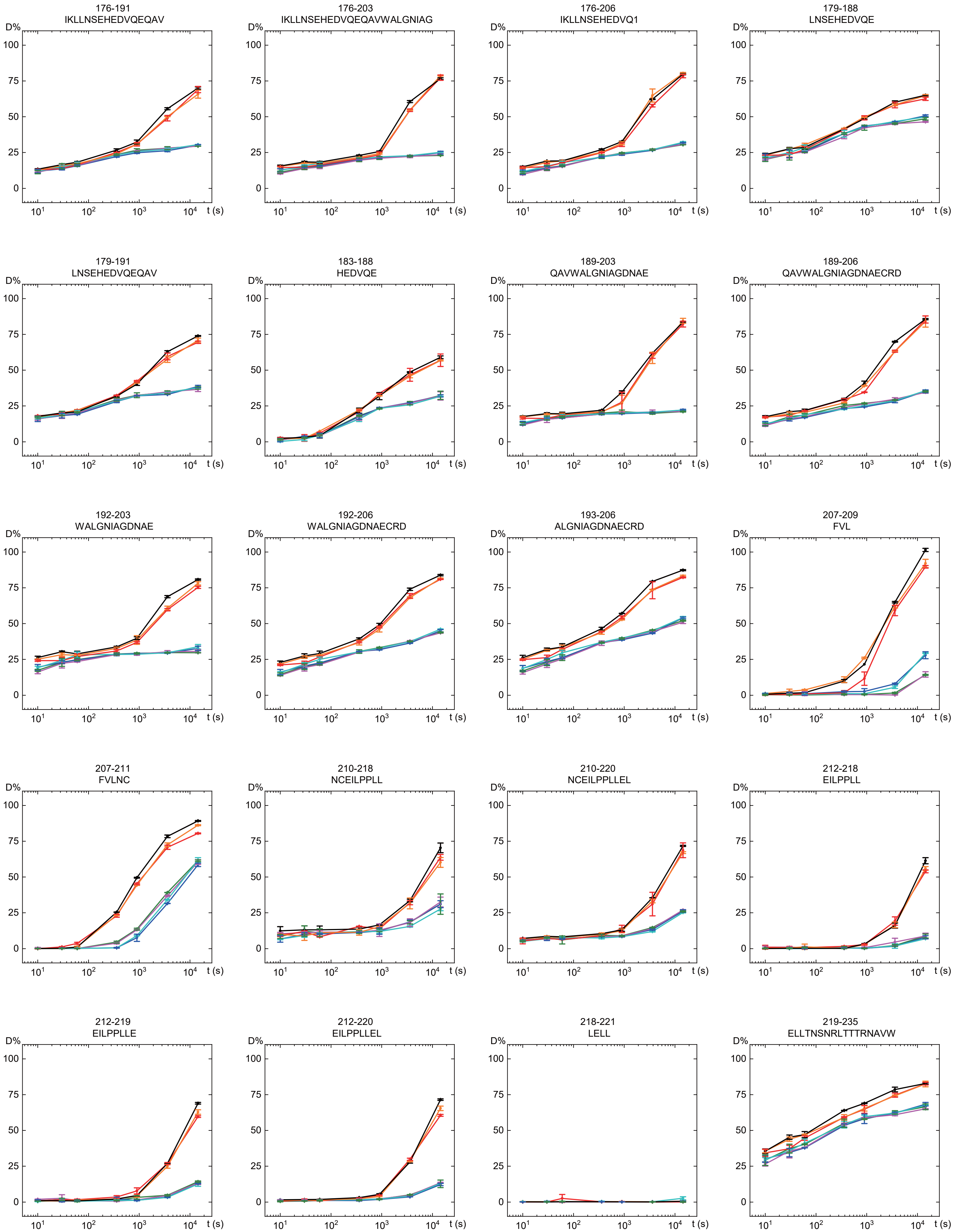

Supplementary Figure S3

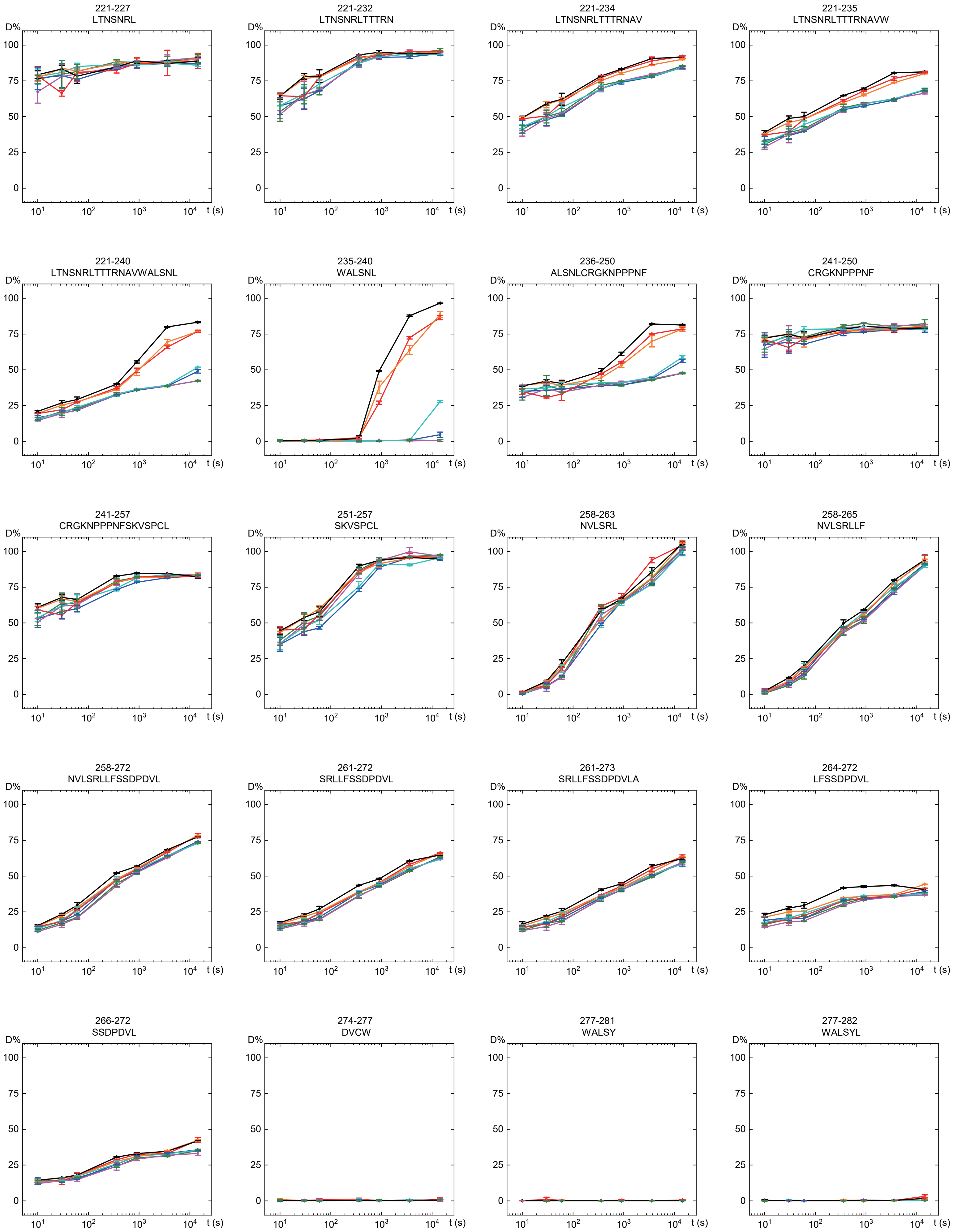

Supplementary Figure S3

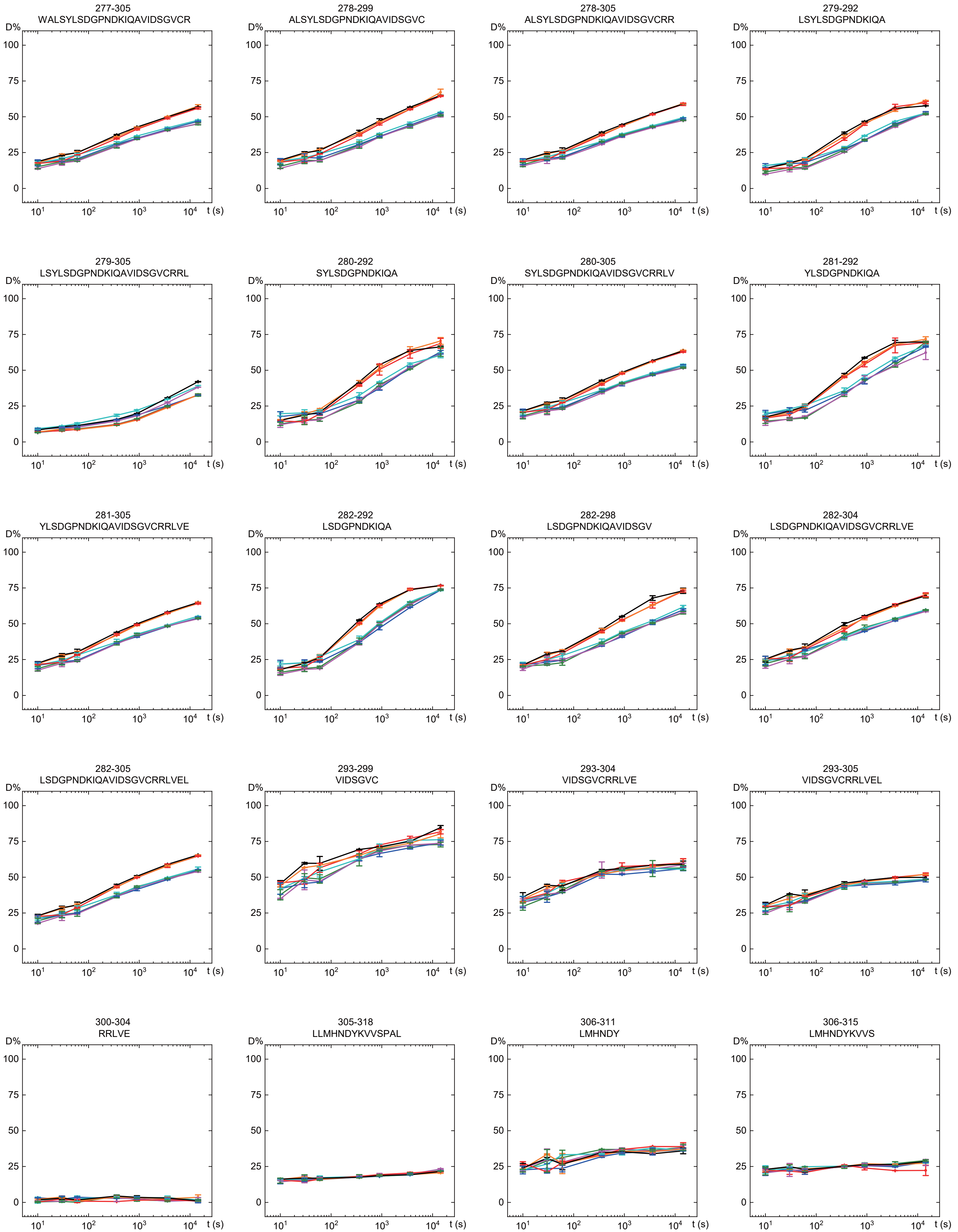

Supplementary Figure S3

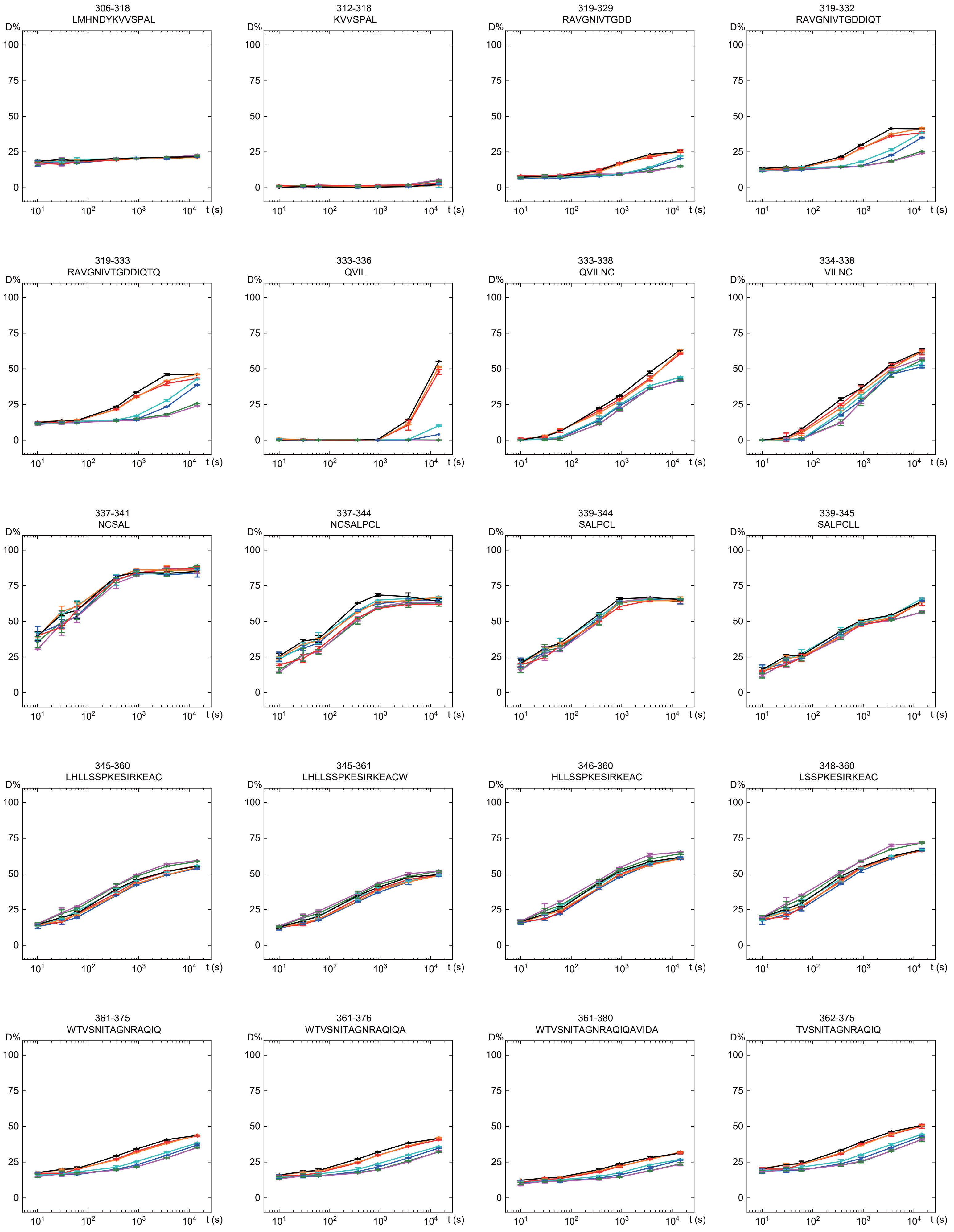

Supplementary Figure S3

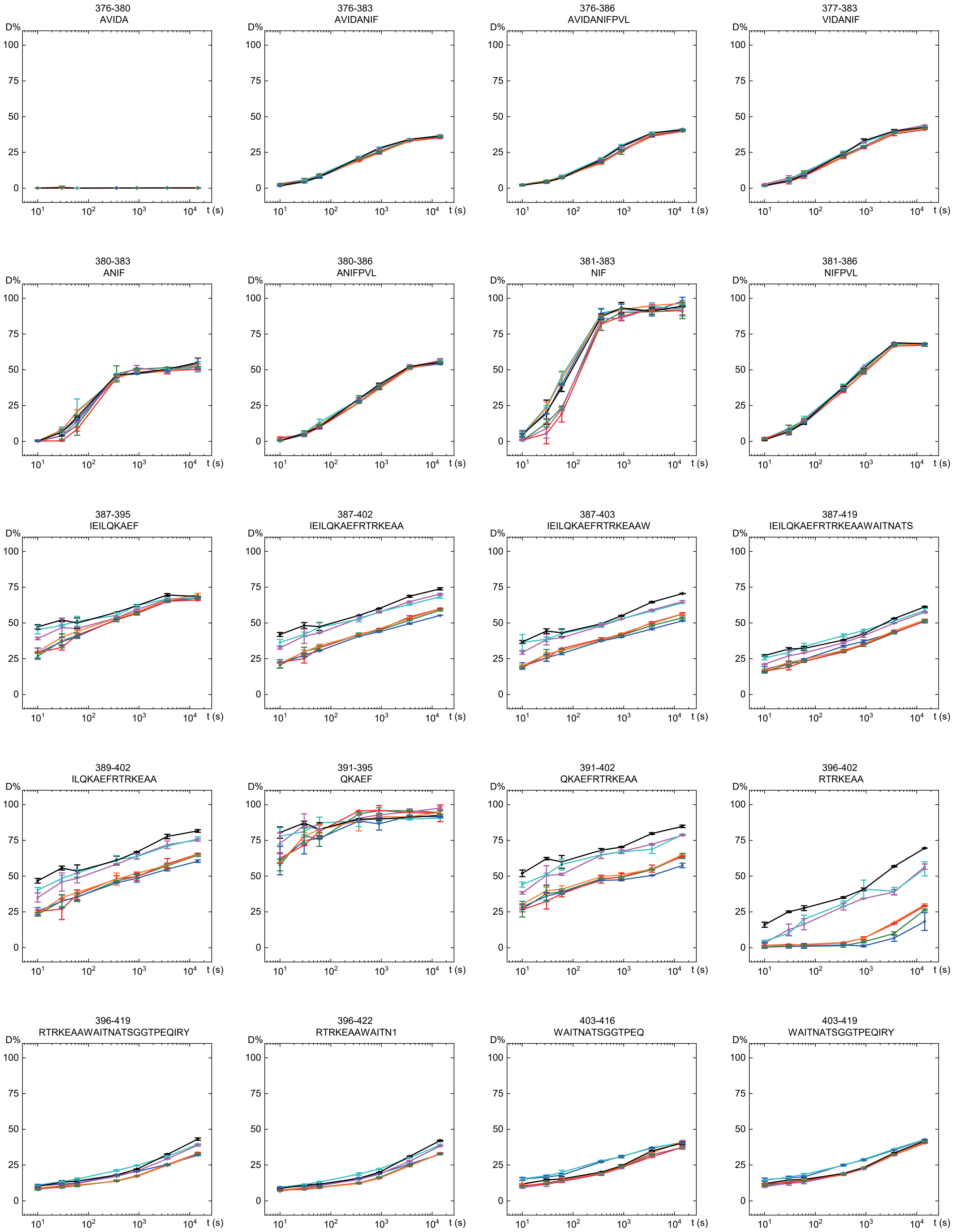

Supplementary Figure S3

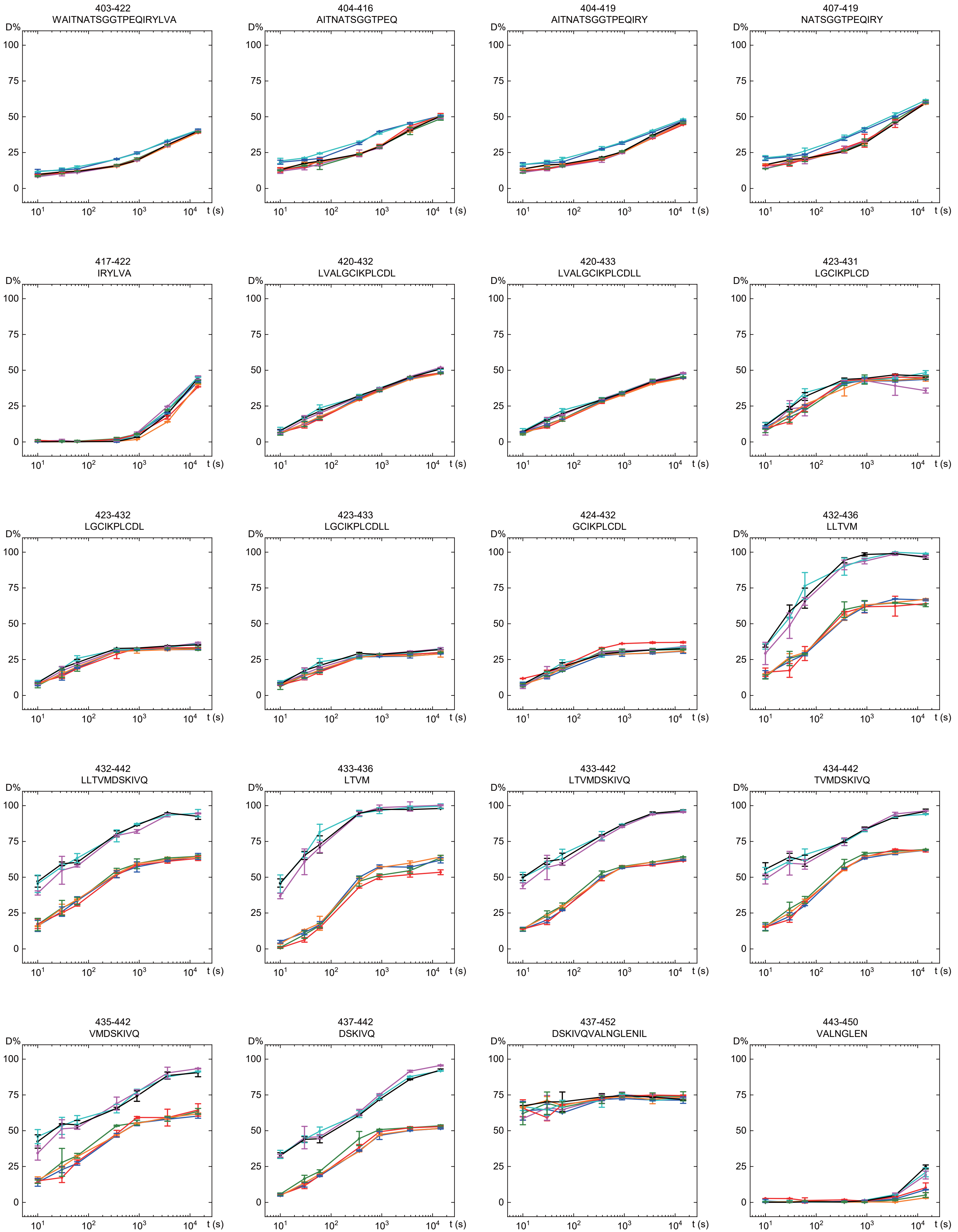

Supplementary Figure S3

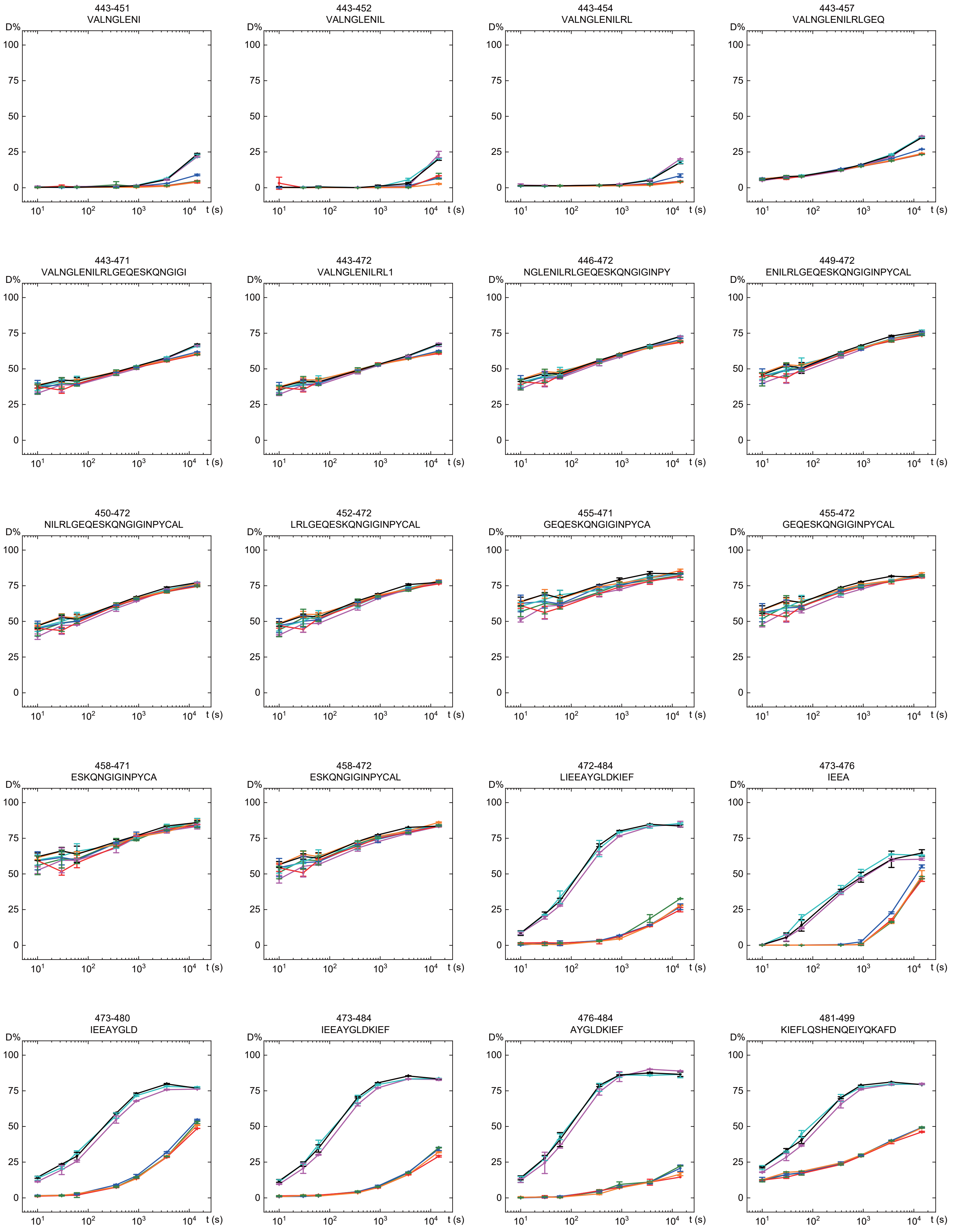

Supplementary Figure S3

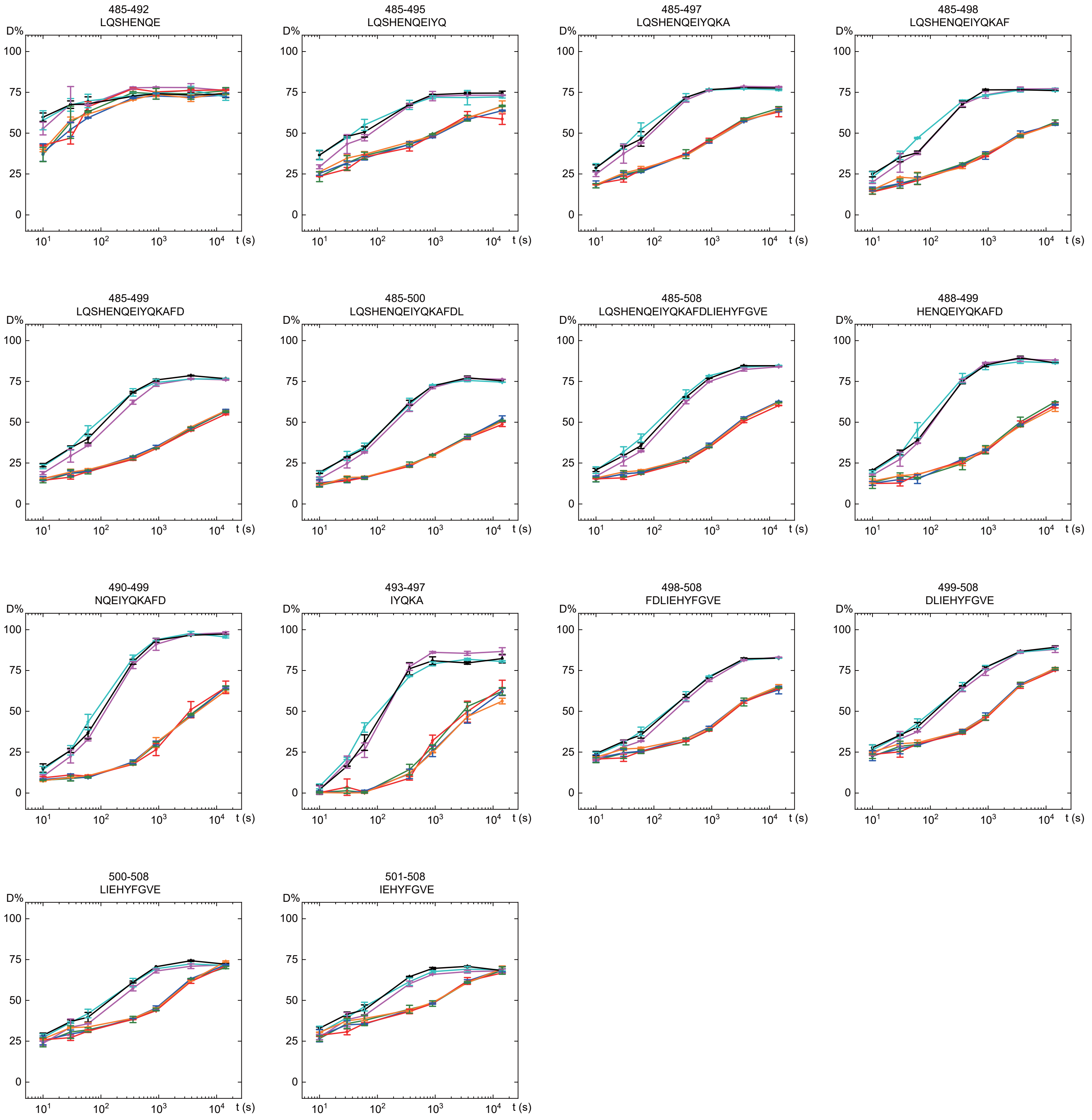

Supplementary Figure S3

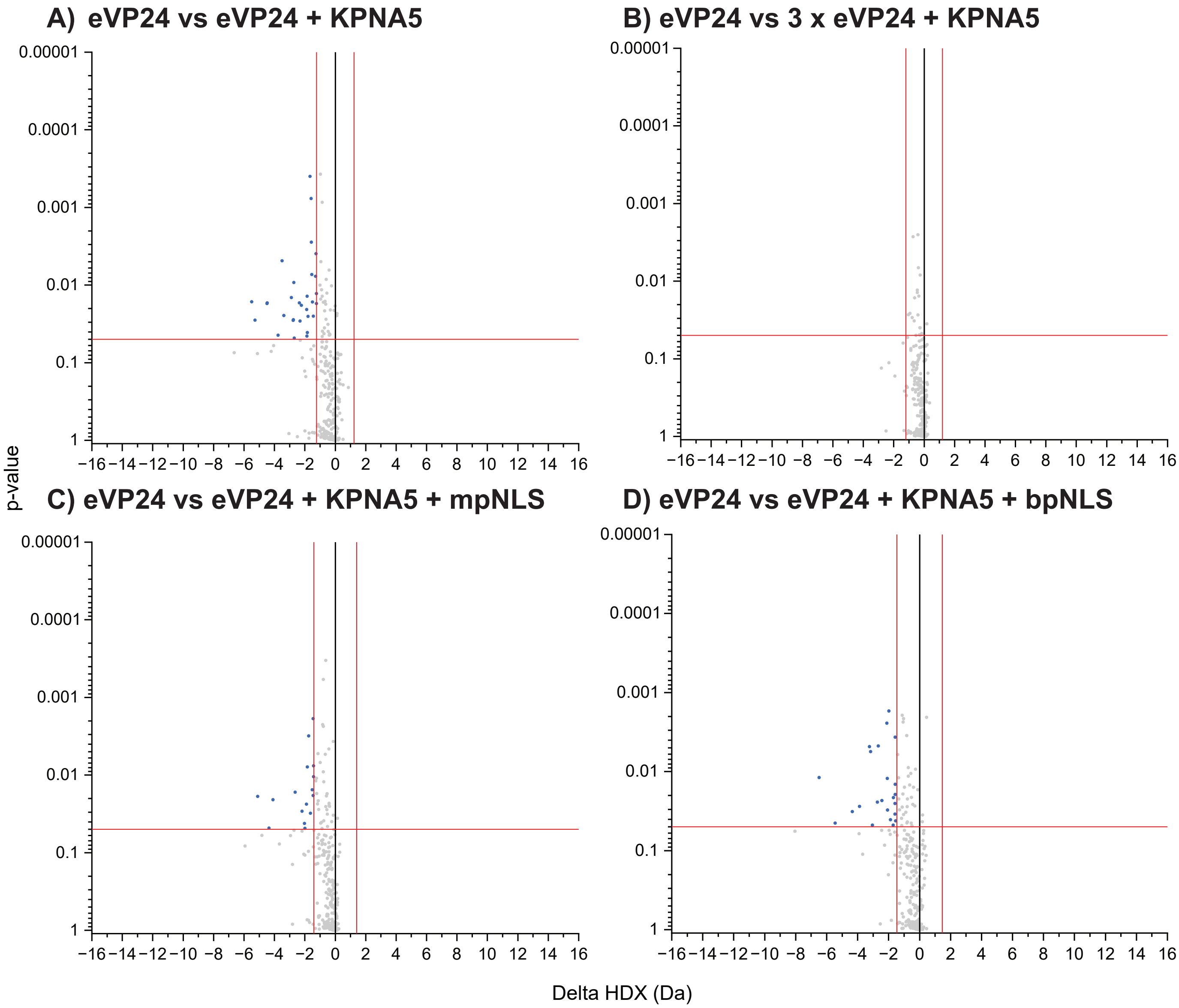

Supplementary Figure S4

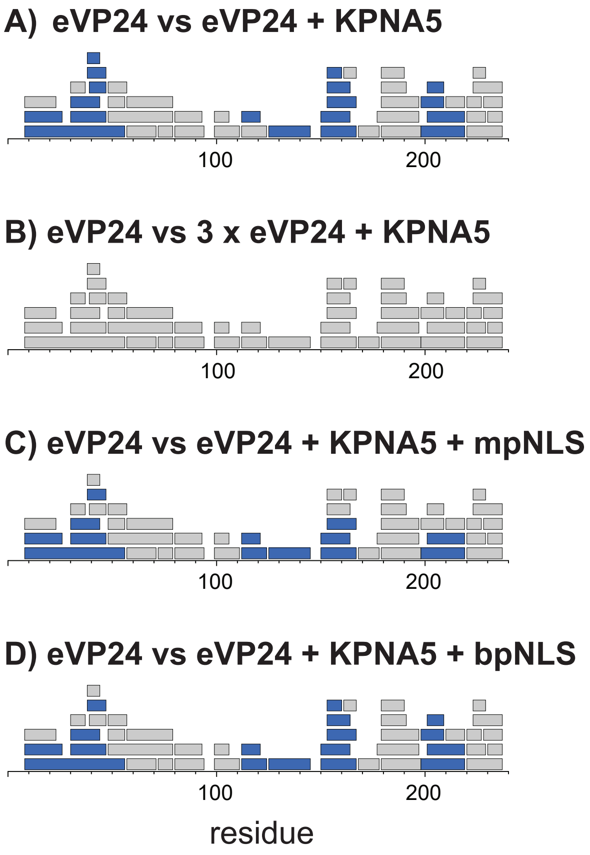

Supplementary Figure S5
